# Metabolism, digestion, and horizontal transfer: potential roles and interaction of symbiotic bacteria in the ladybird beetle *Novius pumilus* and their prey *Icerya aegyptiaca*

**DOI:** 10.1128/spectrum.02955-23

**Published:** 2024-03-18

**Authors:** Xue-Fei Tang, Yi-Fei Sun, Yuan-Sen Liang, Kun-Yu Yang, Pei-Tao Chen, Hao-Sen Li, Yu-Hao Huang, Hong Pang

**Affiliations:** 1 State Key Laboratory of Biocontrol, School of Ecology, Sun Yat-sen University, Shenzhen, China; 2 College of Forestry, Henan Agricultural University, Zhengzhou, China; China Agricultural University, Beijing, China

**Keywords:** *Novius pumilus*, *Icerya aegyptiaca*, symbionts, 16S rRNA, nutrient metabolism, sex determination, digestion, transfer

## Abstract

In this study, we first time sequenced and analyzed the 16S rRNA gene data of predator ladybird beetles *Novius pumilus* and globally distributed invasive pest *Icerya aegyptiaca* at different stages, and combined data with bacterial genome sequences in *N. pumilus* to explored the taxonomic distribution, alpha and beta diversity, differentially abundant bacteria, co-occurrence network, and putative functions of their microbial community. Our finding revealed that *Candidatus* Walczuchella, which exhibited a higher abundance in *I. aegyptiaca*, possessed several genes in essential amino acid biosynthesis and seemed to perform roles in providing nutrients to the host, similar to other obligate symbionts in scale insects. *Lactococcus*, *Serratia*, and *Pseudomonas*, more abundant in *N. pumilus*, were predicted to have genes related to hydrocarbon, fatty acids, and chitin degradation, which may assist their hosts in digesting the wax shell covering the scale insects. Notably, our result showed that *Lactococcus* had relatively higher abundances in adults and eggs compared to other stages in *N. pumilus*, indicating potential vertical transmission. Additionally, we found that *Arsenophonus*, known to influence sex ratios in whitefly and wasp, may also function in *I. aegyptiaca*, probably by influencing nutrient metabolism as it similarly had many genes corresponding to vitamin B and essential amino acid biosynthesis. Also, we observed a potential horizontal transfer of *Arsenophonus* between the scale insect and its predator, with a relatively high abundance in the ladybirds compared to other bacteria from the scale insects.

**IMPORTANCE:**

The composition and dynamic changes of microbiome in different developmental stages of ladybird beetles *Novius pumilus* with its prey *Icerya aegyptiaca* were detected. We found that *Candidatus* Walczuchella, abundant in *I. aegyptiaca,* probably provide nutrients to their host based on their amino acid biosynthesis-related genes. Abundant symbionts in *N. pumilus*, including *Lactococcus*, *Serratia*, and *Pseudophonus*, may help the host digest the scale insects with their hydrocarbon, fatty acid, and chitin degrading-related genes. A key endosymbiont *Arsenophonus* may play potential roles in the nutrient metabolisms and sex determination in *I. aegyptiaca*, and is possibly transferred from the scale insect to the predator.

## INTRODUCTION

Symbionts in insects play a complicated role, which have been approved to function in diet digestion (1), nutrients provision (2, 3), detoxification (4), and influencing the pathogens for transovarial transmission (5). For example, the flavobacterial endosymbiont in the giant scale insect *Llaveia axin axin* (Hemiptera: Coccoidea: Monophlebidae) is supposed to biosynthesize essential amino acid for the host, which constitutes their principal contribution in this relationship (3). Moreover, bacterial symbionts of insects can interact across trophic levels, and influence the dynamics among plants, their hosts’ competitors, and natural enemies (6–9).

Several studies have focused on the bacteria acting between aphid-feeding ladybirds and aphids (9, 10). For example, the research exploring the association between aphidophagous ladybird beetles and aphids has revealed that a free-living strain of aphid symbiont, *Serratia symbiotica*, which can protect its aphid host from various environmental stresses, exhibits a non-negative impact on aphidophagous ladybirds, and in turn uses the ladybird as the medium looking for the next host (9). Nevertheless, investigations on the relationship between coccidophagous (scale-feeding) ladybirds and coccids have rarely been studied.

*Icerya aegyptiaca* (Douglas) (Hemiptera: Coccoidea: Monophlebidae) is a globally distributed invasive pest, attacking at least 123 plant species and secreting honeydew harmful for the plant (11). As a member of Iceryini*, I. aegyptiaca* has developed hydrophobicity wax shells, primarily of hydrocarbons, alcohols, N-acids, and hydroxyl acids composed (12). It undergoes three immature instars before reaching the female stage and four before the male stage (13). However, their male is rarely observed, and female have developed sex determination mechanisms that can either self-fertilize or mate with males (14). *Novius* Mulsant, 1846 (= *Rodolia* Mulsant, 1850), a genus of ladybird beetles distributed worldwide (Coleoptera, Coccinellidae), is obligate in feeding on scale pests (mainly *Icerya*), such as *Novius pumilus* (Weise, 1892) (= *Rodolia pumila* Weise, 1892). They have been used in biocontrol for various *Icerya* species, including *I. aegyptiaca*, *Icerya purchasi*, and *Icerya seychellarum* (15, 16). Their life stages include four larval stages, pupa, and adult (17). Genomic and transcriptomic resources of *N. pumilus* and *I. aegyptiaca* have shed light on the issues like prey adaptation of ladybirds, evolution of structures, reproductive systems, and symbiotic relationships in the scale insects (18, 19). However, the characteristics and potential interactions of their endosymbionts remain unclear.

Previous studies have confirmed changes in the microbiome during the life stages of various insects, like silkworms (20), butterflies (21), leaf beetles (22), and mosquitoes (23). It has been suggested that the functional profile of microbiome may develop with their host (24), and the insect-microbe associations are under dynamic changes (25). In *Diaphorina citri* (Hemiptera: Liviidae), by studying the 16S rRNA data from different development stages, it has been hypothesized that certain genera, like *Profftella*, are vertically inherited (26). Therefore, studying symbiotic bacteria in different development stages of *I. aegyptiaca* and *N. pumilus* may help explore the potential functions during the development of hosts in both the predator and prey.

In the present study, we sequenced the 16S rRNA V3-V4 region of *N. pumilus* and *I. aegyptiaca*, uncovering the composition and potential interactions of the microbiome during different developmental stages of ladybirds and their prey. We explored the taxonomic distribution, alpha and beta diversity, differentially abundant bacteria, co-occurrence network, and putative functions of their microbial community. Additionally, the integration of gene annotations of bacterial sequences from the raw genome assembly of *N. pumilus* (18) provided further evidence to confirm the functions of endosymbionts in the scale insects and the ladybird beetles.

## RESULTS

### General features and characterization of microbial community

A total of 15,445,644 of reads were achieved from Illumina HiSeq platform (Supplementary Material 2: Table S1). After denoising and filtering using DADA2 (27) in Quantitative Insights Into Microbial Ecology version 2 (QIIME2) (28), 8,782–88,081 features for *I. aegyptiaca* and 33,753–116,766 features for *N. pumilus* were retained (Supplementary Material 2: Table S1).

**Fig 1:**
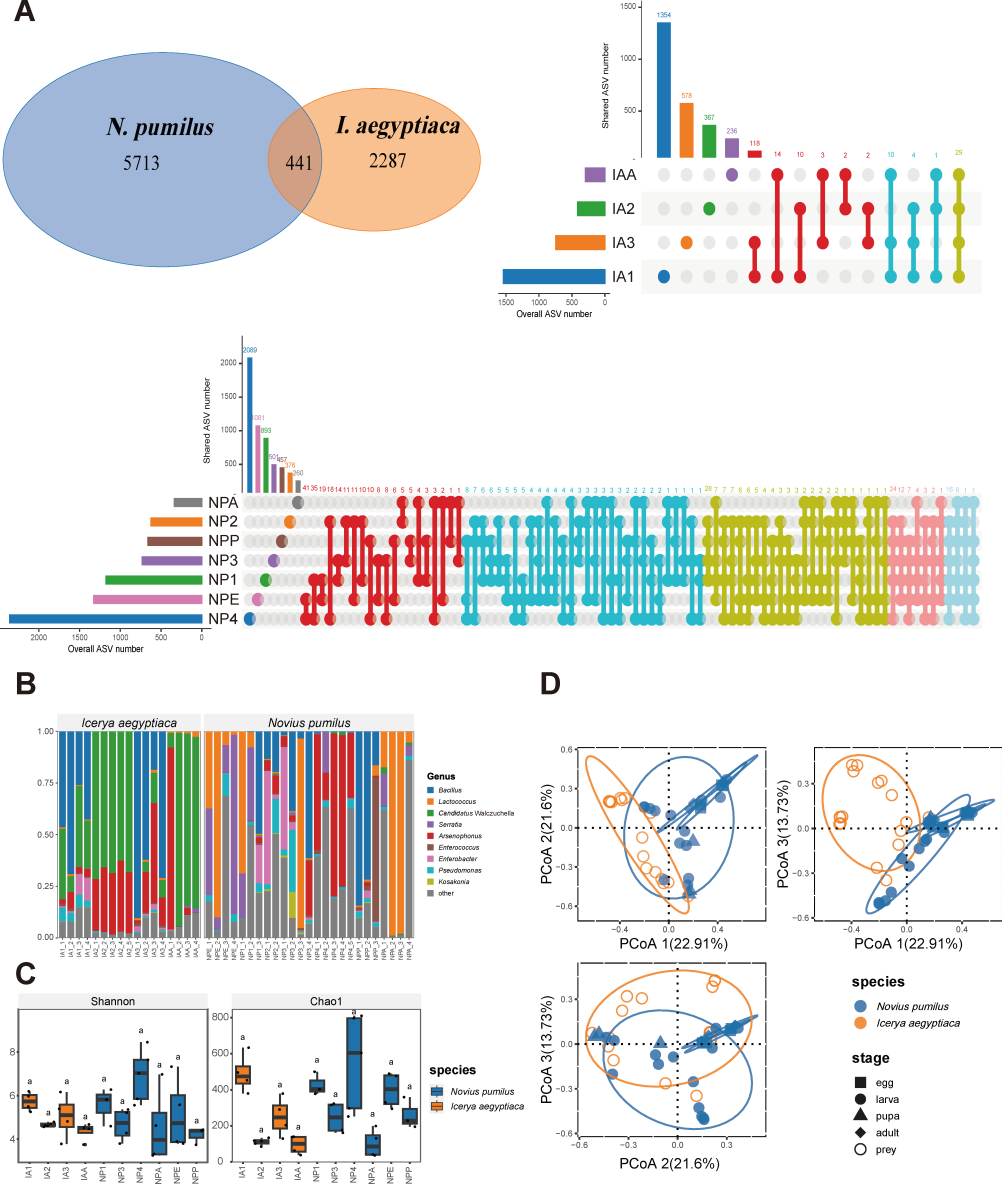
Comparative analyses of the 16S rRNA data of *Novius pumilus* and *Icerya aegyptiaca*. (**A**) Venn diagram and UpSet diagram displaying shared and unique ASVs between *N. pumilus* and *I. aegyptiaca* of different instar stages in each species. (**B**) Genus-level taxonomic composition in each sample. (**C**) Scattered boxplots of Shannon and Chao1 indices of different instar stages in *N. pumilus* and *I. aegyptiaca*. The 2nd instar larva stage of *N. pumilus* was excluded from the analysis due to only two samples. (**D**) Principal coordinate analysis (PCoA) based on Bray-Curtis distance. The ellipses represent the confidence intervals, and the confidence intervals of pupa of *N. pumilus* are not displayed due to samples less than four. Abbreviation in the group names: IA, *I. aegyptiaca*; NP, *N. pumilus*; E, egg stage; 1, first instar nymph/larvae stage; 2, second instar nymph/larvae stage; 3, third instar nymph/larvae stage; 4, fourth instar larvae stage; P, pupa stage; A, adult stage.

In total, 8,527 amplicon sequence variants (ASVs) were identified, consisting 5,713 specific to *N. pumilus* and 2,287 specific to *I. aegyptiaca*, and 441 shared between the two species (Fig. 1A). Twenty-nine ASVs annotated as *Candidatus* Walczuchella and *Arsenophonus* were shared among all different ages of *I. aegyptiaca* (Fig. 1A). No ASV was shared among all ages of *N. pumilus*, while 26 ASVs were shared among at least five stages, and 48 were shared among the egg and adult, which mainly include *Lactococcus*, *Serratia*, *Pseudomonas*, and *Elizabethkingia*. (Fig. 1A).

Seven thousand four hundred and twenty-three ASVs were assigned to known bacteria. In *N. pumilus*, the top 3 most abundant phyla were Firmicutes (48.690%), Proteobacteria (42.779%), and Bacteroidota (2.599%) (Supplementary Material 1: Fig. S1). In *I. aegyptiaca*, the top 3 most abundant phyla were Bacteroidota (40.997%), Firmicutes (33.042%), and Proteobacteria (24.061%) (Supplementary Material 1: Fig. S1). The most abundant genera in *N. pumilus* were *Lactococcus* (27.75%), *Bacillus* (16.84%), *Serratia* (11.10%), *Arsenophonus* (10.53%), *Enterobacter* (4.08%), *Pseudomonas* (3.88%), and *Enterococcus* (3.11%) (Fig. 1B). The most abundant genera in *I. aegyptiaca* were *Candidatus* Walczuchella (39.94%), *Bacillus* (31.35%), *Arsenophonus* (15.53%), *Enterobacter* (2.35%), and *Pseudomonas* (2.32%) (Fig. 1B). *Bacillus* exhibited relatively high abundance in both species, whereas *Lactococcus* mainly appeared in *N. pumilus. Arsenophonus* was a dominant population in the second to third instar nymphs and adults of *I. aegyptiaca* (Fig. 1B), and with a relatively high abundance in the fourth instar larvae of *N. pumilus*. It was also detected in several scattered samples of other development stages in *N. pumilus*, but with relatively low abundances (Fig. 1B). The ladybird beetle predators displayed higher diversities of endobacteria than the prey, with higher percentages of bacteria out of the top 9 genera.

### Diversity analysis

The rarefaction curve of each sample approached a plateau, indicating that the sequencing depth was sufficient for accurately inferring the abundance of the bacterial community (Supplementary Material 1: Fig. S2). All samples were normalized to 8,782 sequences corresponding to the lowest number of sequences in Supplementary Material 2: Table S1. The bacterial alpha diversity of each sample was calculated using Shannon and Chao1 indices, and the significance levels between different species or stages were tested. The results revealed no significant difference in Shannon index (*P*-value = 0.710) between the two species (Supplementary Material 1: Fig. S3). However, the Chao1 index of *N. pumilus* was significantly higher than that of *I. aegyptiaca* (*P*-value = 0.047) (Supplementary Material 1: Fig. S3), indicating a higher richness in the predator. Both Shannon and Chao1 indexes showed some difference among the instar stages, but no statistical significance was found (*q*-value: 0.089–1.000) (Fig. 1C). This suggests that there were no significant differences in the diversity and richness among each development stage.

The community dissimilarity was measured using Bray-Curtis distance and visualized using principal coordinate analysis (PCoA) (Fig. 1D). The scale insect and ladybird can be roughly clustered into two parts. The larva and pupa samples of *N. pumilus* had a larger overlapping region with *I. aegyptiaca*, indicating the prey may exert a more substantial influence on the early age of the predator (Fig. 1D). The egg and adult of *N. pumilus* shared a larger overlapping region, with coexistence and probable maternal transmission of some bacteria, such as *Lactococcus* (Fig. 1B and D).

### Differential abundance in bacteria of different species and development stages

**Fig 2:**
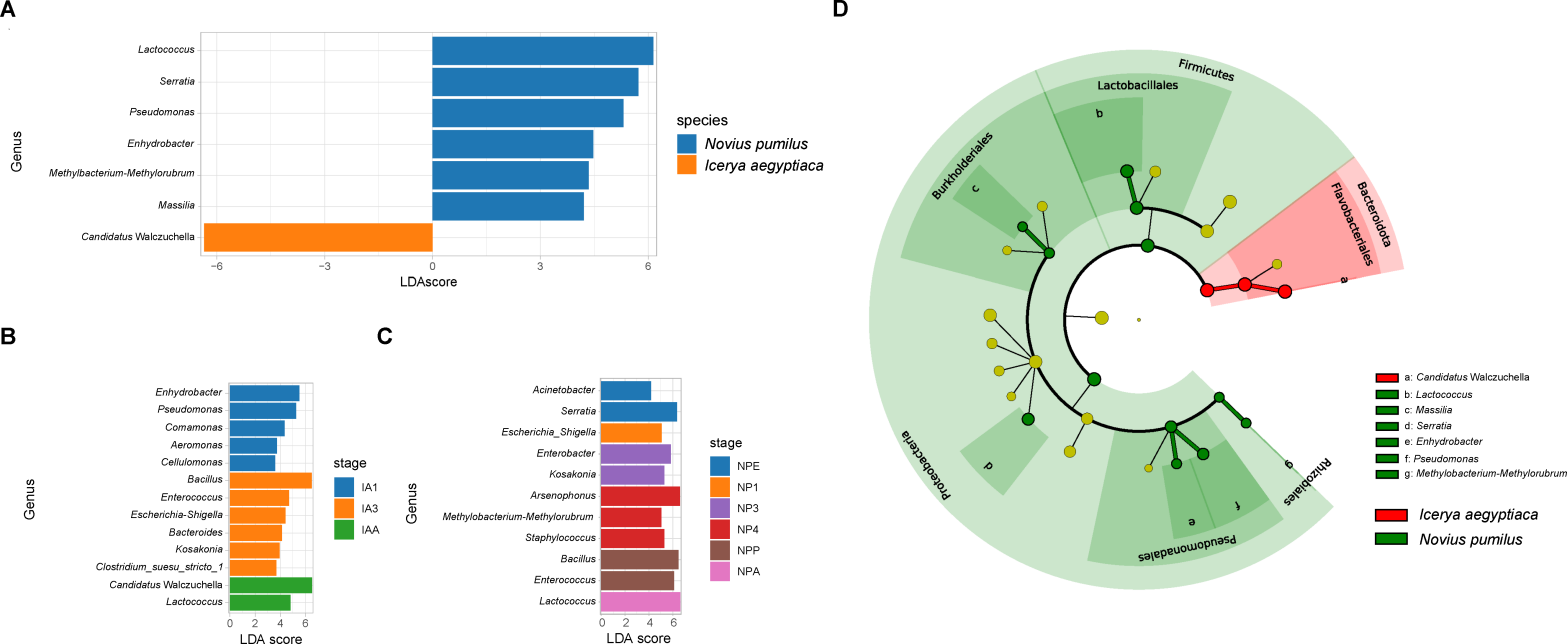
LEfSe results of significantly overrepresented genera between *Novius pumilus* and *Icerya aegyptiaca* or different stages in each species. (**A**) The significantly overrepresented genera for *N. pumilus* of *I. aegyptiaca*. (**B**) The significantly overrepresented genera for different stages of *I. aegyptiaca*. (**C**) The significantly overrepresented genera for different stages of *N. pumilus*. The second instar larva stage was excluded from the analysis due to only two samples. (**D**) The LEfSe cladogram for genera, orders, and phyla of different species. Abbreviation in the group names: IA, *I. aegyptiaca*; NP, *N. pumilus*; E, egg stage; 1, first instar nymph/larvae stage; 2, second instar nymph/larvae stage; 3, third instar nymph/larvae stage; 4, fourth instar larvae stage; P, pupa stage; A, adult stage.

Linear discriminant analysis (LDA) effect size (LEfSe) analysis (29) was conducted on the MicrobiomeAnalyst website (https://www.microbiomeanalyst.ca/) (30) to identify bacteria with significantly different abundances between different species and ages. The LEfSe result confirmed the significantly higher abundance of the genus *Candidatus* Walczuchella in *I. aegyptiaca* and *Lactococcus*, *Serratia*, *Pseudomonas*, *Enhydrobacter*, *Methylobacterium*, and *Massilia* in *N. pumilus* (Fig. 2A and D). Regarding the different development stages of *I. aegyptiaca*, *Enterobacter*, *Pseudomonas*, and *Comamonas* had a higher abundance in the first instar nymph of *I. aegyptiaca*, while *Bacillus*, *Enterococcus* and *Escherichia-Shigella* were more abundant in the third instar nymph (Fig. 2B), and *Candidatus* Walczuchella and *Lactococcus* for the adult (Fig. 2B). *Candidatus* Walczuchella and *Arsenophonus* displayed high abundances in the second instar stage of *I. aegyptiaca*, but no significant differences were detected by LEfSe. For the different development ages of *N. pumilus*, higher abundances were observed in *Serratia* and *Acinetobacter* in the egg stages, *Escherichia-Shigella* in the first instar larva, and *Kosakonia* and *Enterobacter* in the third instar larva (Fig. 2C). The fourth instar larva stage showed significantly higher abundances of *Arsenophonus*, *Methylobacterium-Methylorubrum* and *Staphylococcus* (Fig. 2C). *Bacillus* and *Enterococcus* were dominant in the pupa stage (Fig. 2C). *Lactococcus* was identified as high-abundance bacteria in the adult stage of *N. pumilus* (Fig. 2C).

### Co-occurrence network

**Fig 3:**
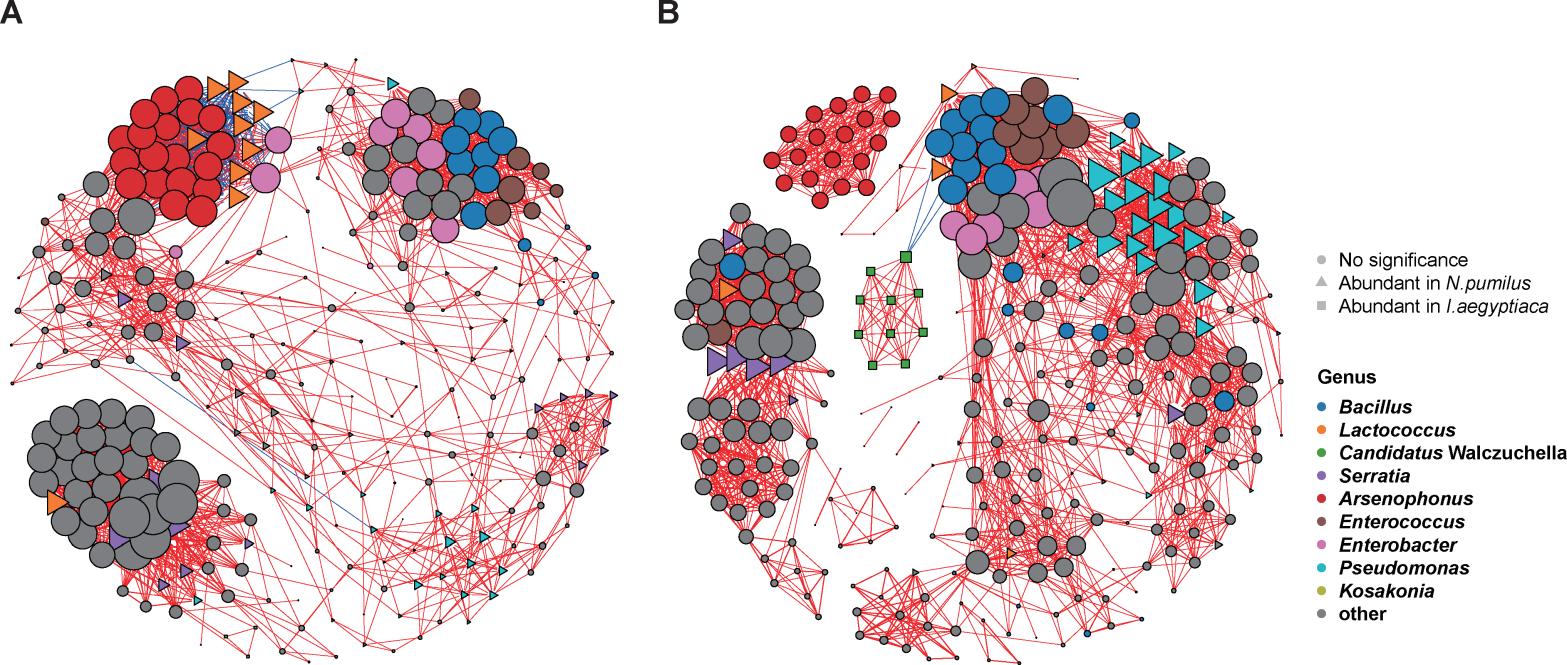
The co-occurrence networks of the bacteria in *Novius pumilus* and *Icerya aegyptiaca*. (**A**) The network of *N. pumilus*. (**B**) The network of *I. aegyptiaca*. The ASVs belonging to the top 10 genera with the highest abundances were colored based on taxonomy. Red edges indicated positive correlations (Spearman’s correlation coefficients >0.6 and *P* < 0.001), while blue edges indicated negative correlations (Spearman’s correlation coefficients <−0.6 and *P* < 0.001). The size of each node positively correlated to the degree of this node. The nodes were shaped based on the LEfSe results.

Co-occurrence networks were established for the top 300 ASVs with the highest abundance in *N. pumilus* and *I. aegyptiaca* based on correlation. The network of *N. pumilus* comprised 286 nodes and 2,257 edges (Fig. 3A; Supplementary Material 2: Table S3). The network of *I. aegyptiaca* consisted of 298 nodes and 2,694 edges (Fig. 3B; Supplementary Material 2: Table S3). The negative edges, average path length, network diameter, and global clustering coefficient of the network of *N. pumilus* were larger, and the total edges, connectance, and average degree of the network of *I. aegyptiaca* were larger, indicating the microbial network of the scale insect might be more complex than the ladybird, while the adjacent nodes were connected better in *N. pumilus* based on the clustering coefficient.

The co-occurrence networks of different stages of each species were also constructed using the top 300 ASVs of the feature table, with other criteria remained unchanged (Supplementary Material 1: Fig. S4 to S13; Supplementary Material 2: Table S3). For *N. pumilus*, the adult stage had the fewest nodes and edges among all ages (except the second instar larva). The first instar larva stage followed by the pupa stage showed the highest number of edges, connectance, and average degree, indicating more complex endosymbiont interactions in these two stages. For *I. aegyptiaca*, the number of nodes, edges, and average degree of the second nymph stage were apparently lower than other stages, displaying the lowest microbial network complexity. The network of third nymph stage was the most complicated as the number of edges, connectance, and average degree were the largest.

In *N. pumilus*, *Arsenophonus*, *Lactococcus*, *Bacillus*, *Enterobacter*, and *Serratia* had relatively high degrees. *Bacillus*, *Enterobacter*, and *Enterococcus* showed mainly positive interactions with each other, while *Lactococcus*, *Pseudomonas*, *Enterobacter*, and *Arsenophonus* displayed relative amounts of negative interactions with each other. In *I. aegyptiaca*, *Arsenophonus*, *Bacillus*, *Candidatus* Walczuchella*, Pseudomonas*, and *Enterococcus* possessed high degrees. *Arsenophonus* and *Candidatus* Walczuchella exhibited positive interactions within themselves. Meanwhile, *Candidatus* Walczuchella had negative interactions with *Bacillus*.

### Function analysis

**Fig 4:**
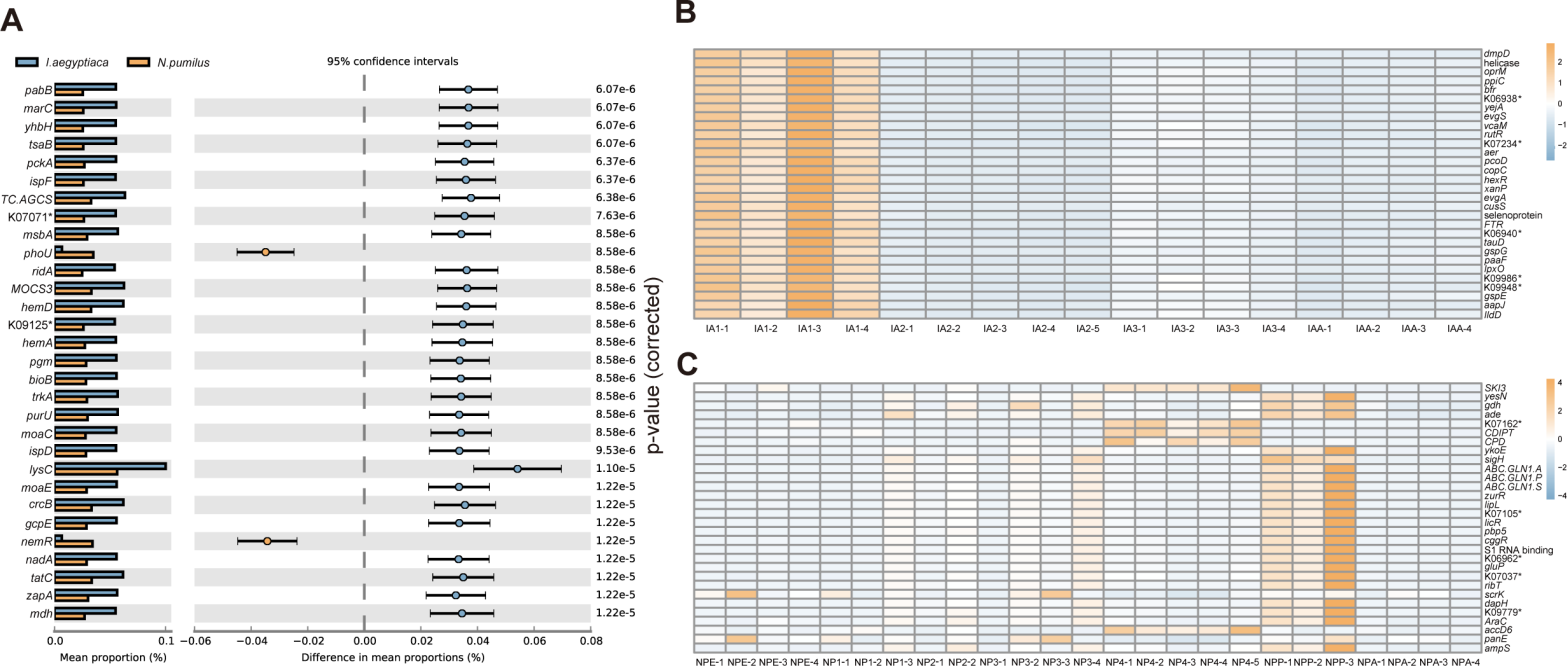
The STAMP result of different functions in bacteria of *Novius pumilus* and *Icerya aegyptiaca* of different stages in each species. (**A**) The top 30 KEGG Orthologs significantly higher in *N. pumilus* or *I. aegyptiaca*. (**B**) Heatmaps that displayed the abundance of top 30 KEGG Orthologs recognized by STAMP of different instar stages of *N. pumilus*. (**C**) Heatmaps that displayed the abundance of top 30 KEGG Orthologs recognized by STAMP of different instar stages of *I. aegyptiaca*. * displayed uncharacterized genes in KEGG database. Abbreviation in the group names: IA, *I. aegyptiaca*; NP, *N. pumilus*; E, egg stage; 1, first instar nymph/larvae stage; 2, second instar nymph/larvae stage; 3, third instar nymph/larvae stage; 4, fourth instar larvae stage; P, pupa stage; A, adult stage.

Phylogenetic Investigation of Communities by Reconstruction of Unobserved States 2 (PICRUSt2) (31) was employed to predict the Kyoto Encyclopedia of Genes and Genomes (KEGG) Orthologs, and the abundance of function genes in different species or stages were compared with STAMP (32). In *N. pumilus*, genes mainly related to genetic information processing (K16137: *nemR*), transport (K02037: *pstC*, K02038: *pstA*, K02030: *ABC.PA.S*), and metabolism (K01426: *amiE*, K03823: *pat*, K00574: *cfa*) were predicted to be significantly higher (*P* < 0.001) (Fig. 4A; Supplementary Material 1: Fig. S14). In *I. aegyptiaca*, genes related to transport (K03310: *TC.AGCS*, K03118: *tatC*, K06199: *crcB*), metabolism (K01665: *pabB*, K01835: *pgm*, K01770: *ispF*, K017192: *hemD*), and genetic information processing (K14742: *tsaB*, K05808: *yhbH*, K09888: *zapA*) were predicted to be significantly higher (*P* < 0.001) (Fig. 4A, Supplementary Material 1: Fig. S14). For *I. aegyptiaca*, genes mainly related to metabolism (K03594: *bfr*, K12979: *lpxO*, K00101: *lldD*), signaling and cellular processes (K07245: *pcoD*, K16345: *xanP*), and transport (K13893: *yejA*, K18893: *vcaM*) exhibited higher abundances in the first nymph stage (Fig. 4B). Regarding *N. pumilus* with different stages, the pupa stage displayed higher abundances of genes related to genetic information process (K03091: *sigH*, K03491: *licR*) (Fig. 4C). Furthermore, metabolism-correlated genes (K00999: *CDIPT*, K18472: *accD6*, K07752: *CPD*) had higher abundances in the fourth instar larvae (Fig. 4C).

**Fig 5:**
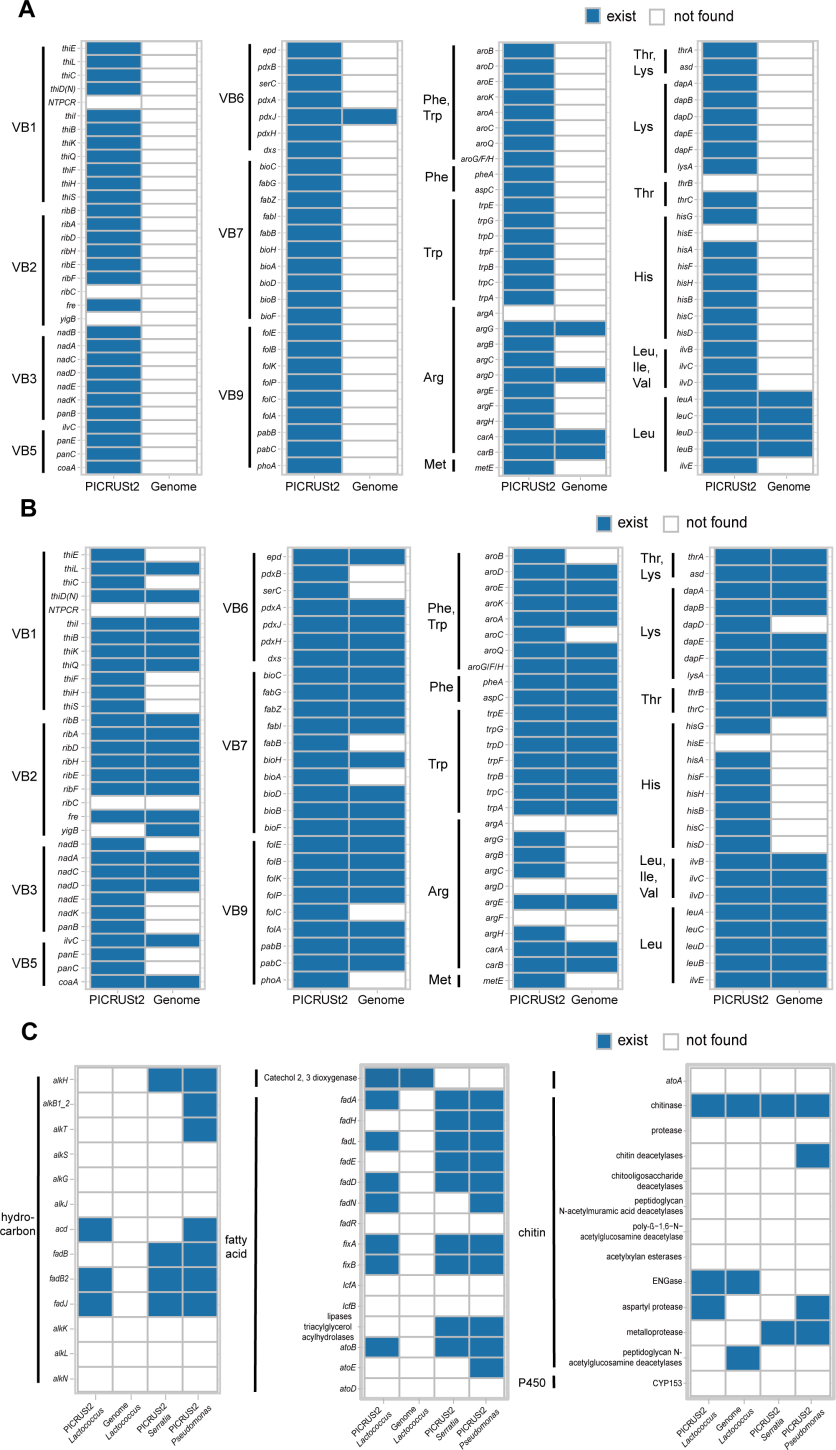
Genes involved in nutrient biosynthesis and hydrocarbon, fatty acid, and chitin degradation pathways. (**A**) Gene existences of vitamin B and amino acids biosynthesis pathways in *Candidatus* Walczuchella. (**B**) Gene existences of vitamin B and amino acid biosynthesis pathways in *Arsenophonus*. (**C**) Gene existences of hydrocarbon, fatty acid, and chitin degradation in *Lactococcus*, *Serratia*, and *Pseudomonas*.

We also identified 51 genes related to the amino acid biosynthesis in *Candidatus* Walczuchella, which were more abundant in *I. aegyptiaca*. The only missing three genes were *argA* for arginine biosynthesis, *thrB* for threonine biosynthesis, and *hisE* for histidine biosynthesis (Fig. 5A). Moreover, our results revealed 56 genes associated with vitamin B biosynthesis (Fig. 5A).

Previous studies have confirmed the capabilities of *Arsenophonus* to provide hosts with vitamin B and essential amino acids that are absent in their food (33–35). Based on these research findings, we found 56 vitamin B biosynthesis genes and 49 essential amino acid biosynthesis genes in PICRUSt2 results. The missing genes were *NTPCR* for thiamine biosynthesis, *ribC* and *yigB* for riboflavin biosynthesis, *argA*, *argD,* and *argF* for arginine biosynthesis, and *hisE* for histidine biosynthesis (Fig. 5B).

*Lactococcus*, *Serratia*, and *Pseudomonas* were found to have high abundances in several stages of *N. pumilus*, and these bacteria were previously reported as wax-degrading bacteria in scale insects (36–39). Considering the characteristic wax shell covering the scale insects, we focused on exploring genes and enzymes possibly related to prey digestion, particularly those related to hydrocarbon and fatty acid degradation. Our results identified the presence of several genes and enzymes, including *fixB* (K03522), *fixA* (K03521), *fadD* (K01897), *atoB* (K00626), *alkH* (K00128), catechol 2, 3 dioxygenase gene (K07104), catalase (K03781), and esterase *LipB* (EC:3.1.1.1), (Fig. 5C; Supplementary Material 2: Table S6). STAMP results indicated a portion of these genes had significantly higher abundances in *N. pumilus* than in *I. aegyptiaca*, including *fadH* (*q*-value: 1.11e − 3), *atoB* (*q*-value: 2.98e-3), *acd* (*q*-value: 2.14e − 3), *fadJ* (*q*-value: 1.56e − 3), *fadB* (*q*-value: 2.51e − 3), *atoE* (*q*-value: 9.45e − 3), *fadE* (*q*-value: 8.02e − 3) (Supplementary Material 1: Fig. S15). Additionally, genes and enzymes corresponded to the degradation of chitin also existed, including chitinase (K01183), endo-β-*N*-acetylglucosaminidase (ENGase, K01227), and chitin deacetylases (K01452) (Supplementary Material 2: Table S4). The STAMP result confirmed the higher abundance of ENGase and chitinase in *N. pumilus* compared to *I. aegyptiaca* (*q*-value: 0.014/8.89e − 4) (Supplementary Material 1: Fig. S15).

### Analysis of bacterial genome sequences from *N. pumilus*

In total, eight species were recognized from 42 bacterial genome sequences from *N. pumilus*, including *Lactococcus lactis*, *Lactococcus allomyrinae*, *Arsenophonus nasoniae*, *Candidatus* Walczuchella monophlebidarum, *Serratia marcescens*, *Sodalis* sp. and *Salmonella* sp. (Supplementary Material 2: Table S4). Totally 6,924 proteins were predicted using Prokka (40).

In the annotation of sequences of *Candidatus* Walczuchella, several genes related to essential amid acid biosynthesis were found, including *argD* and *argG*, required for tryptophan and phenylalanine biosynthesis, *carA* and *carB* for arginine biosynthesis, and *leuA*, *leuB*, *leuC,* and *leuD* for leucine biosynthesis (Fig. 5A; Supplementary Material 2: Table S5). Among the genes related to vitamin B biosynthesis, only *pdxJ* for pyridoxine biosynthesis was present (Fig. 5A; Supplementary Material 2: Table S5). We found *A. nasoniae* exhibited a multitude of genes involved in the biosynthesis of vitamin and amid acids, which contained almost complete riboflavin (vitamin B2) pathway except *ribC*. But it lacked key genes for the complete synthesis of other vitamin B members, like *thiE* for thiamine, *nadB*, *nadE,* and *nadK* for nicotinic acid, and *bioA* for biotin (Fig. 5B; Supplementary Material 2: Table S5) (34, 35). Thirty-five genes responding to the essential amino acid biosynthesis were also found. However, genes taking part in the biosynthesis of arginine and histidine were missing, while those correlated to leucine biosynthesis were all present (Fig. 5B; Supplementary Material 2: Table S5).

In the sequences of *L. lactis* and *L. allomyrinae*, we found genes related to the degradation of chitin, including chitinase, ENGase, and peptidoglycan N-acetylglucosamine deacetylases (Fig. 5C; Supplementary Material 2: Table S5). But none of these genes were found in the sequence of *S. marcescens*.

## DISCUSSION

### Roles of symbiotic bacteria in nutrient metabolisms in *I. aegyptiaca*

Our result revealed that most species with differential abundances in the scale insect belonged to Bacteroidota, mostly *Candidatus* Walczuchella (Flavobacteriales). For the Monophlebidae family, the obligate symbionts were considered to be flavobacteria , as reported in *I. purchasi* (41, 42). The primary roles of obligate symbionts (the primary symbiont) in most scale insects include the synthesis of essential nutrients that are lacking in their hosts’ diets (41). Furthermore, previous studies have indicated that *Candidatus* Walczuchella, as an obligate symbiont, displayed important roles in the biosynthesis of essential amino acids in at least three Monophlebidae scale insects, including *I. purchasi*, *L. axin axin,* and *Drosicha piniola* (3, 41–44). In addition, the co-occurrence network showed that *Candidatus* Walczuchella had few correlations with other symbionts in *I. aegyptiaca*. We speculate that it may reside in specialized bacteriocytes, as it was in *L. axin axin* and flavobacteria in other scale insects (3, 42, 43, 45, 46). In the function prediction and bacterial genome sequences, as expected, various genes correlated to the biosynthesis of essential amino acids were found in *Candidatus* Walczuchella. Pathways involved in the biosynthesis of phenylalanine, tryptophan, and leucine were nearly complete. However, due to the incompleteness of bacterial genome sequences, not all genes could be confirmed in both PICRUSt2 results and the genome data. It is uncertain whether these genes actually exist in our strains.

Among different stages, the strains of *Candidatus* Walczuchella were more abundant in the first and third instar nymphs of *I. aegyptiaca*, suggesting a probably higher nutrition demand during the nymph stage compared to the adult stage. STAMP results showed that metabolism-related genes were significantly more abundant in the first and third nymph stage but less in the adult stage, which was also an indication. The complete genome of *Walczuchella monophlebidarum* from the giant scale insect *L. axin axin* was found to be strongly reduced (3). Several genes involved in the biosynthesis of certain amino acids have been lost or pseudogenized, like *aroE*, *aspC*, *hisC*, *hisD*, *dapE*, *dapF*, and *ilvE*, which were also absent in the bacterial genome sequences in our study. The absent genes in *Walczuchella* could be found in the enterobacterial symbiont of *L. axin axin*, which fulfills the flavobacterium’s functions (3). Both obligate and facultative symbionts establish a stable coexistence in the host by this way (3). Similarly, in the bacterial sequences of *Arsenophonus* in our study, the lost genes mentioned above were all present except *hisC* and *hisD*. Hence, *Walczuchella* in *I. aegyptiaca* may also need to coordinate with other endobacteria.

### Roles of symbiotic bacteria in prey digestion in *N. pumilus*

Symbiotic bacteria are widespread in the ladybird beetle populations and can play vital characters in food digestion (47–49). For example, several cellulolytic bacteria have been found in pollen-fed ladybird *Micraspis discolor* and probably participate in pollen digestion (49). But studies on endosymbionts in those predators assisting to digest their foods are still in rare cases.

In our study, we found significantly higher abundances of *Lactococcus*, *Serratia*, and *Pseudomonas* in *N. pumilus* than *I. aegyptiaca*. Previous reports have confirmed *Serratia* and *Pseudomonas* as wax-degrading bacteria isolated from the scale insects (36–39). In addition, the chitinolytic system of *L. lactis* has been studied and found to contain a chitinase (β-1,4-poly-N-acetyl glucosaminidase; EC 3.2.1.14) and a chitin-binding domain (50). It was also reported that this bacterium is involved in the digestion of the termite *Reticulitermes chinensis* Snyder (51). Thus, it seems possible that bacteria from these three genera contribute to the digestion of the scale prey and its wax shell in the ladybird.

The wax shell of *I. purchasi* and another scale insect, the mealybug *Phenacoccus solenopsis*, are composed of hydrocarbons, alcohols, N-acids, hydroxyl acids, fatty acids, aromatic derivatives, and esters (12, 52). Therefore, we investigated potential wax and cuticle degrading-related genes in strains belonging to *Lactococcus*, *Serratia*, and *Pseudomonas*. The results of PICRUSt2 revealed the presence of genes responding in alkane and fatty acids degradation, like *fixB*, *fixA*, *fadD*, *atoB*, *alkH* (53–55). A portion of these genes possessed significantly higher abundances in *N. pumilus* than in *I. aegyptiaca*, including *fadH*, *atoB*, *acd*, *fadB*, *atoE*, and *fadE*, indicating their potential higher requirements in *N. pumilus*. The roles of *fad* regulon in fatty acid degradation in *Escherichia coli* and *Bacillus subtilis* have been well studied (54). Although some differences were present in their pathway, but *fadE*, *fadH*, *fadB*, and *fadA* were both necessary, and all of these three genes were present in our function prediction of *Lactococcus*, *Serratia,* and *Psuedomonas*. However, these genes were absent in the bacterial genome sequences of *L. lactis* and *S. marcescens*, on account of the real missing or the incompleteness of the bacterial genome sequences. In the bacterial genome sequences of *L. lactis* and *S. marcescens*, catechol 2, 3 dioxygenase gene was found, which was also presented in the PICRUSt2 results. This gene is considered to be a key degrading gene in hydrocarbon-degrading bacterial strains isolated from the polluted soil (56). Therefore, the enriched endosymbionts in *N. pumilus*, like *Lactococcus*, *Serratia*, and *Pseudomonas*, appear to possess the abilities to degrade the scale insects’ wax shell, which needs further verification.

We also observed the presence of several chitin degrading-related genes in the bacterial genome sequences of *L. lactis* and *S. marcescens*, including ENGase, chitinase, and *pgdA* (57, 58). Both ENGase and chitinase were presented in the PICRUSt2 result and bacterial genome sequences of *L. lactis*, and ENGase was also presented in the PICRUSt2 results of *Serratia* and *Pseudomonas*. STAMP result confirmed the higher abundance of ENGase and chitinase in *N. pumilus* compared to *I. aegyptiaca*. Chitin is a significant component of insect cuticles and a barrier to break through (59). Wax-degrading bacteria isolated from the cadavers of the scale insects were also reported to possess the ability of producing chitinase, which can utilize wax and increase the natural mortality of mealybugs (36, 39). Hence, it is probable that endosymbionts in the ladybird can help their hosts digest the prey itself or the wax shell as well.

We observed significantly higher abundances of *Serratia* and *Lactococcus* in the egg and adult stages of *N. pumilus,* respectively, and all three bacteria existed in most stages with varying proportions, indicating that the microbiome exhibits variation in community composition during different developing stages. *Lactococcus* represented relatively high abundances in both egg and adult stages, suggesting it may be maternally inherited. Maternally inherited endobacteria can play critical roles in the evolution and ecology of their hosts (60), and by vertical transfer, bacteria can remain association with the insect host for millions of years (61). Vertically transmitted endosymbionts in insects can play various roles, such as in nutrient supplements, protecting their hosts against natural enemies, and manipulating host reproduction, like *Wolbachia*, *Spiroplasma*, and *Serratia* (62). But the maternal transmission of *Lactococcus* has rare reports. It is possible that *Lactococcus* in the egg of *N. pumilus* may also provide the larva with the initial or enhanced ability to digest their food.

### Possible roles in sex determination and potential horizontal transfer between trophic level of *Arsenophonus*

In the *N. pumilus-I. aegyptiaca* system, a genus named *Arsenophonus* (Proteobacteria: Enterobacteriaceae) was conspicuous, which possessed relatively high abundances in the second instar nymph and adult stages of *I. aegyptiaca*, and the fourth instar larvae stage of *N. pumilus*, though no significance among different stages was detected by LEfSe analysis in *I. aegyptiaca. Arsenophonus* is a group of symbionts distributed in arthropods and plants (63). It has been confirmed to play parts in nutrition metabolism and influence on sex ratio of their hosts (64). The first genome of *Arsenophonus* was that in the wasp *Nasonia vitripennis*, where it inhibits the formation of maternal centrosomes to perform male-killing (65, 66). Genomes of *Arsenophonus* from whitefly indicate its potential role in producing B vitamins (33). Further studies demonstrate that *Arsenophonus* possessed genes related to the biosynthesis of vitamin B and essential amino acids, which may control the sex of hosts via nutrient metabolism (34, 35). In our strains of *Arsenophonus*, we also found the existence of vitamin B and essential amino acid biosynthesis-related genes. Riboflavin, folate, tryptophan, isoleucine, valine, and leucine synthesis pathways were almost complete, and several missing genes may be due to the incompleteness of the sequences or actual loss. The genus *Icerya*, including *I. aegyptiaca*, is hermaphroditism and has an extreme sex bias that males are often rare in the nature (14). Their female is developed from fertilized egg, and the male is from unfertilized egg, somewhat similar to the haplodiploid whitefly. It is possible that endosymbionts in *I. aegyptiaca*, probably *Arsenophonus*, similar to those in whiteflies, may also take part in sex determination by controlling the biosynthesis of nutrients like vitamin B and essential amino acid, because vitamin B are coenzymes for the synthesis and metabolism of protein and lipids, which are used for oogenesis (34). Actually, *I. aegyptiaca* and the whitefly share several commons in the biosynthesis of vitamin B, including the presence of *Arsenophonus,* and horizontally transferred gene *bioD* (19). The presence of similar endosymbionts and horizontal gene transfers reveals a possible connection between these two phenomena.

We also found a significantly higher abundance of *Arsenophonus* in the fourth instar larva stage of *N. pumilus*, but rare in other stages. Several sequences in the bacterial genome sequences of *N. pumilus* were also annotated as *A. nasoniae*, which also demonstrated probably high abundances in the ladybird. The endosymbionts of foods can have residues in the predators (9). But considering the relatively low abundance of other specific symbionts from *I. aegyptiaca*, like *Candidatus* Walczuchella, it is reasonable to predict that *Arsenophonus* in *N. pumilus* was horizontally transferred from *I. aegyptiaca*, rather than from food scraps. Recently, several studies on the system of plant-aphids-ladybirds have found that endosymbionts between trophic levels can be harmful to the predators or establish a nearly neutral relationship with the predators (9, 67). For the transfer among the trophic levels in the scale insects, there were also some reports on the endosymbiont transferred from plants to the scale insect and produce nutrients to its new host (68). However, the transfer between scale insects and their predators has few reports yet. *Arsenophonus* in the co-occurrence network of *N. pumilus* showed a relative high degree and negative connectance with *Lactococcus*, but its exact role in the ladybird is still unknown. If the transfer that we propose is true, the roles of *Arsenophonus* in ladybird beetles deserve to be further studied in the future to help us realize more about the relationships between endosymbionts, prey, and predators.

## MATERIALS AND METHODS

### DNA extraction and sequencing of the V3-V4 region of 16S rRNA

The female adults of *I. aegyptiaca* (IA) were wild collected from the *Litsea monopetala* tree at the south campus of Sun Yat-sen University, Guangzhou, China, in 2022, and subsequently reared on the host plant *L. monopetala*. *N. pumilus* (NP) were also wild collected from *L. monopetala* tree at the south campus of Sun Yat-sen University in 2022 and maintained on those populations of *I. aegyptiaca*. Both populations were maintained for at least 6 months and through at least three generations to control their interactions with each other, excluding interactions with other insects in the field. First, second, and third instar nymphs and female adults of *I. aegyptiaca*, as well as eggs, first to fourth instar larvae, pupae, and female adults of *N. pumilus* were collected in 2022 for the experiments, respectively. To ensure sufficient DNA quantity, three individuals were combined as one sequencing sample and one biological replicate, and three to five biological replicates were set for each stage of each species. The total genomic DNA of each sample was extracted using Bacterial DNA Extraction Mini kit (Mabio). DNA quality and quantity were detected by a Nanodrop 1000 spectrophotometer (Thermo Fisher Scientific, Wilmington, USA). Only DNA samples with a 260:280 ratio ranging from 1.8 to 2.0, together with a 260:230 ratio ranging from 2.0 to 2.5 were retained for sequencing. Because one of the samples of the second instar larva of *N. pumilus* was unqualified when DNA extraction and further sample supplement were influenced by the coronavirus disease 2019 epidemic situation, leading to only two samples, we removed this stage from further diversity and the LEfSe analysis (Supplementary Material 2: Table S1). The V3-V4 region of 16S rRNA was amplified using specific V3 forward primer 338F (5′- ACTCCTACGGGAGGCAGCA-3′) and V4 reverse primer 806R (5′- GGACTACHVGGGTWTCTAAT-3′) (69). Sequencing was performed on the Illumina Nova 6000 platform.

### Identification and taxonomic assignment of ASVs

The qualities of raw reads obtained from Illumina sequencing platform were checked by FastQC program v0.11.9 (https://www.bioinformatics.babraham.ac.uk/projects/fastqc/). Clean reads with high quality were processed using QIIME2 (28). The sequences were denoised and chimera filtered via DADA2 (27) using the standard denoised-paired command. The feature table of ASVs was generated, and taxonomy was assigned in QIIME2 using Naïve Bayes Silva classifiers, which was trained using the fit-classifier-naïve-bayes module with the SILVA database (70) for 16S rRNA (SILVA 138 SSU Ref NR 99 full-length) for our primers. All parameters were set as default values. The taxonomy results of the top 10 genera with the highest abundances were further verified and annotated by performing BLASTN (71) against National Center for Biotechnology Information (NCBI) nucleotide sequence (NT) database online. Specifically, the strain annotated as *Candidatus* Uzinura in the SILVA database was corrected to *Candidatus* Walczuchella based on higher identity (detailed BLAST hits in Supplementary Material 2: Table S2).

### Rarefaction and diversity analysis

Alpha rarefaction curves were generated using QIIME2 “diversity” plugin (28) by randomly subsampling the ASV table. Alpha diversity of the microbial community structure was estimated based on Shannon (72) and Chao1 (73) indices. The significance levels among different species and stages was tested in QIIME2 “diversity” plugin using Kruskal-Wallis test, and *P*-values were adjusted using the Benjamini and Hochberg correction method. The beta index was measured using Bray-Curtis distance metrics and visualized in PCoA (74).

### LEfSe analysis

The LEfSe (29) method was used to identify the features most likely responsible for explaining differences between species and stages. LEfSe function in Marker-gene Data Profiling (MDP) modules on the website MicrobiomeAnalyst (http://www.microbiomeanalyst.ca/) (30) was used. The ASV table, taxonomy file, and metadata file were input in the MDP module. Data filtering and data normalization keep the default selections as recommended in Chong et al. (75). For LEfSe analysis, the taxa were considered significant with the default setting as recommended: the false discovery rate (FDR)-adjusted *P*-value <0.1 and linear discriminant analysis (LDA score) >2.0 (75).

### Co-occurrence network analysis

To investigate the relationship of core microbial taxa in *I. aegyptiaca* and *N. pumilus*, the top 300 ASVs were used to construct the co-occurrence networks of different species and stages by weighted correlation network analysis (WGCNA) (76), igraph (77), and qgraph (78) in R 4.2.1, which were used in Yu et al. (79), Zhang et al. (80), and Jiao et al. (81). The step was the same as described in Jiao et al. (81). In detail, robust correlations with Spearman’s correlation coefficients >0.6 or <−0.6 and *P* < 0.001 were retained to construct networks. Since there were only two biological repeats for the second instar larva of *N. pumilus*, which could not be calculated by WGCNA, they were removed from the stage-specific analysis.

### Function analysis

Microbial community functions were predicted by the PICRUSt2 (31). We used the STAMP statistical tool (32) to judge the significant KEGG Orthologs. Statistical comparisons between two species and among different stages were performed using Welch’s *t*-test and analysis of variance, respectively (32). Storey’s FDR was employed for multiple test corrections in all tests.

To gain insights into the functions of key bacteria identified in the above analyses, we focused on the biosynthetic pathways of vitamin B and essential amino acids, along with the genes and pathways to degrade the components of the wax, which are vital in hemipteran insects or digestion of them. The information on vitamin B and essential amino acids in *Arsenophonus* was summarized from Santos-Garcia et al. (33), Wang et al. (34), and Zhu et al. (35). Genes related to essential amino acids in *Candidatus* Walczuchella were referenced from Rosas-Pérez et al. (3). Enzymes related to hydrocarbon, fatty acids, and chitin degradation were considered to function in wax and cuticle degradation based on the components (12, 52), which were summarized from Sabirova et al. (53), Schneiker et al. (82), Fujita et al. (54), Aragunde et al. (57), Pavoncello et al. (55), Salunkhe et al. (36), Arunkumar et al. (37), Xi et al. (58), Karthika et al. (56), and Van Bogaert et al. (83).

### Bacterial genome sequence analysis of *N. pumilus*

To further corroborate the functions predicted by PICRUSt2, we performed taxonomic and functional annotations for the bacterial sequences obtained from the raw genome assembly of *N. pumilus* in Tang et al. (18), which can represent a part of the bacterial genomes in the *I. aegyptiaca-N. pumilus* system.

The open reading frames were called and annotated using Prokka (40). Amino acid sequences generated by Prokka were then annotated using a modified FunAnnotate “annotate” pipeline (https://github.com/nextgenusfs/funannotate) as described in Tang et al. (18). For taxonomic identification, we employed BLASTN (84) against the local NCBI NT database and MEGAN6 (85).

## Data Availability

The 16S data were deposited in the NCBI BioProject: PRJNA995433. The bacterial genome sequences were obtained from the NCBI BioProject: PRJNA626074.

## SUPPLEMENTAL MATERIAL

Supplemental figures spectrum.02955-23-s0001.docx Fig. S1 to S15.

Supplemental tables spectrum.02955-23-s0002.xlsx Tables S1 to S6.

ASM does not own the copyrights to Supplemental Material that may be linked to, or accessed through, an article. The authors have granted ASM a non-exclusive, world-wide license to publish the Supplemental Material files. Please contact the corresponding author directly for reuse.
