# Supplementary material for: Metabolism, digestion, and horizontal transfer: potential roles and interaction of symbiotic bacteria in the ladybird beetle Novius pumilus and their prey Icerya aegyptiaca
Source: Microbiol Spectr. 2024 Mar 18;12(5):e02955-23. doi: 10.1128/spectrum.02955-23 (PMC11064573; doi:10.1128/spectrum.02955-23)
Supplement: Supplemental figures — Fig. S1 to S15. [file spectrum.02955-23-s0001.docx]

Supplementary Material

**Metabolism, digestion and horizontal transfer: potential roles and interaction of symbiotic bacteria in the ladybird beetle *Novius pumilus* and their prey *Icerya aegyptiaca***

**Xue-Fei Tang1, 2†, Yi-Fei Sun1†, Yuan-Sen Liang1, Kun-Yu Yang1, Pei-Tao Chen1, Hao-Sen Li1, Yu-Hao Huang1*, Hong Pang1****

***Author for Correspondence:**

**Hong Pang, State Key Laboratory of Biocontrol, School of Ecology, Sun Yat-sen University, Shenzhen 518107, China.**

**Email: lsshpang@mail.sysu.edu.cn**

**Yu-Hao Huang, State Key Laboratory of Biocontrol, School of Ecology, Sun Yat-sen University, Shenzhen 518107, China.**

**Email: huangyh45@mail2.sysu.edu.cn**


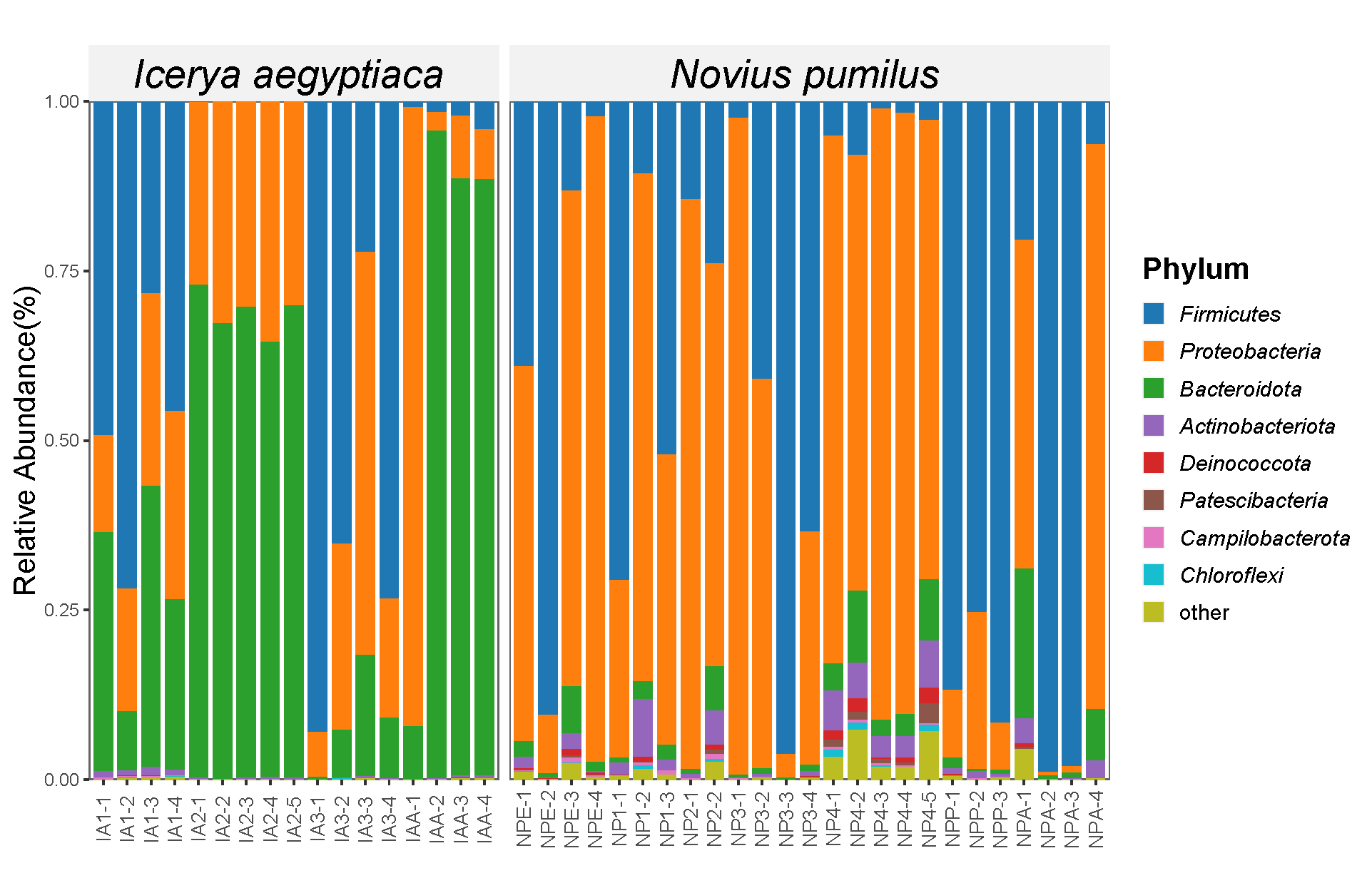


Fig. S1 Phylum-level taxonomic composition in each sample. Abbreviation in the group names: IA: *I. aegyptiaca*; NP: *N. pumilus*; E: egg stage; 1: first instar nymph/larvae stage; 2: second instar nymph/larvae stage; 3: third instar nymph/larvae stage; 4: fourth instar larvae stage; P: pupa stage; A: adult stage.


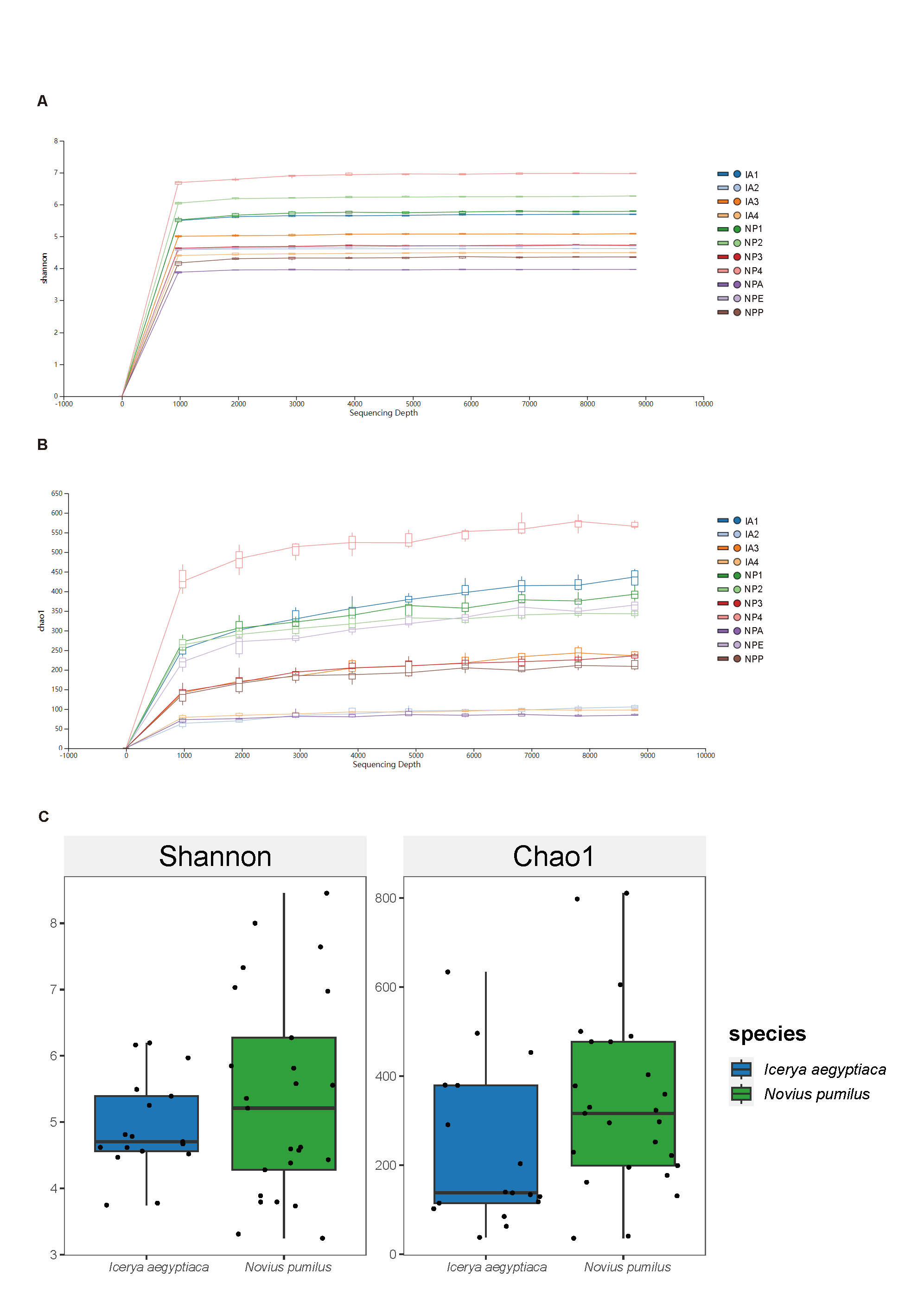


Fig. S2 Rarefaction curves of different stages of *I. aegyptiaca* and *N. pumilus*. (A) Rarefaction curve of Shannon index of different stages. (B) Rarefaction curve of Chao1 index of different stages. Abbreviation in the group names: IA: *I. aegyptiaca*; NP: *N. pumilus*; E: egg stage; 1: first instar nymph/larvae stage; 2: second instar nymph/larvae stage; 3: third instar nymph/larvae stage; 4: fourth instar larvae stage; P: pupa stage; A: adult stage.


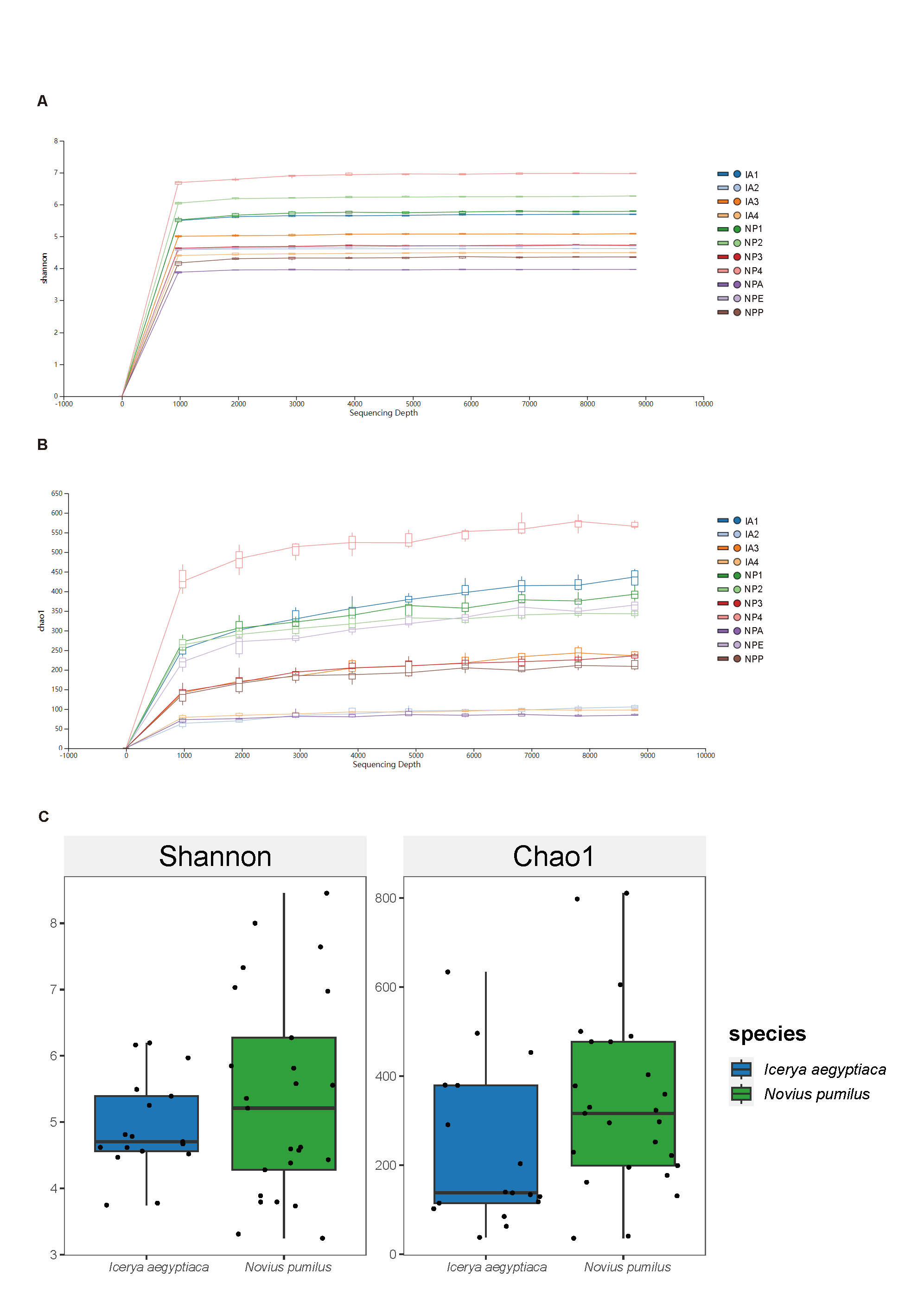


Fig. S3 Scattered boxplots of Shannon and Chao1 index of different species.


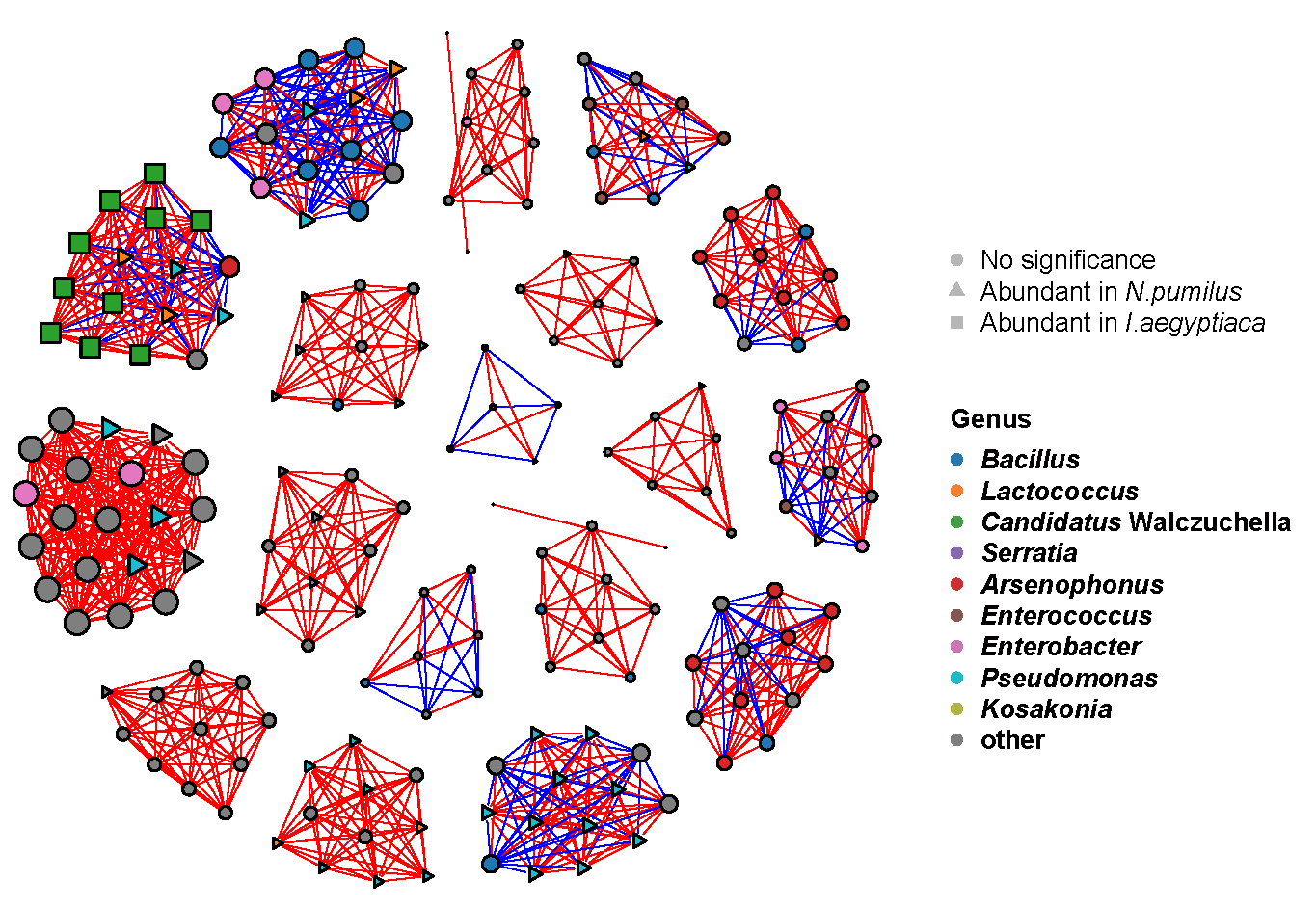


Fig. S4 The co-occurrence networks of the bacteria in the 1^st^ instar nymph stage of *Icerya aegyptiaca*.


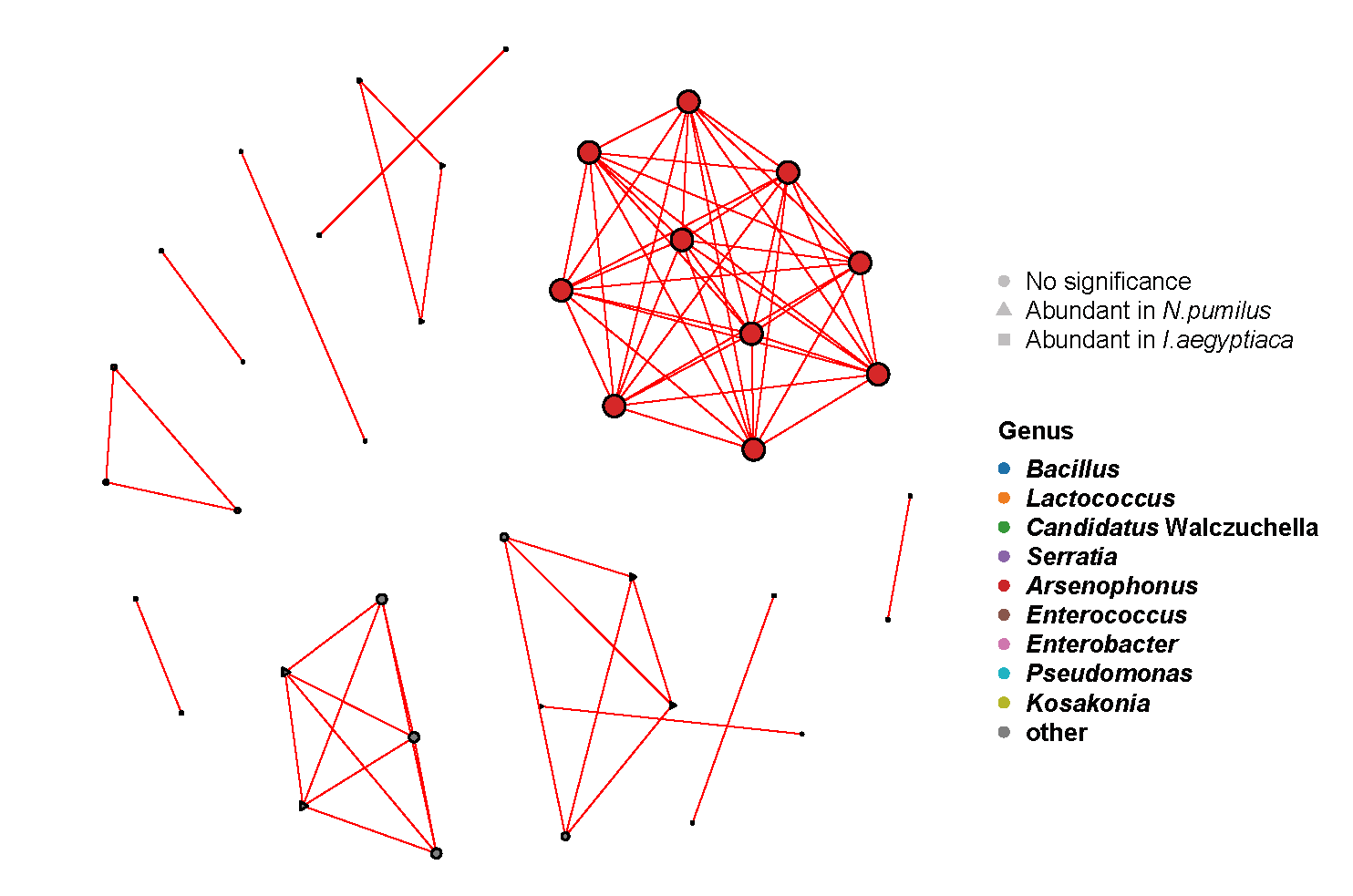


Fig. S5 The co-occurrence networks of the bacteria in the 2^nd^ instar nymph stage of *Icerya aegyptiaca*.


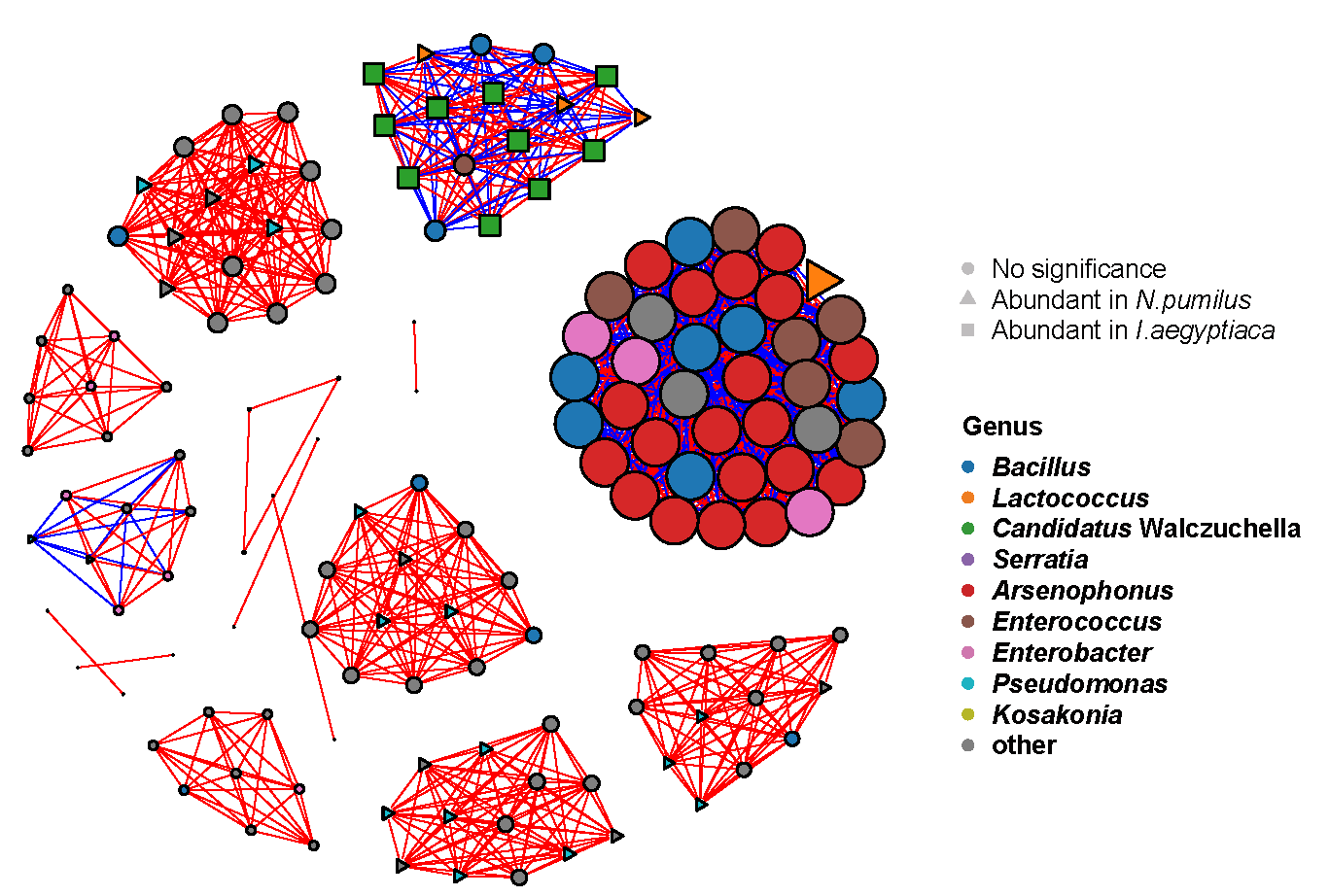


Fig. S6 The co-occurrence networks of the bacteria in the 3^rd^ instar nymph stage of *Icerya aegyptiaca*.


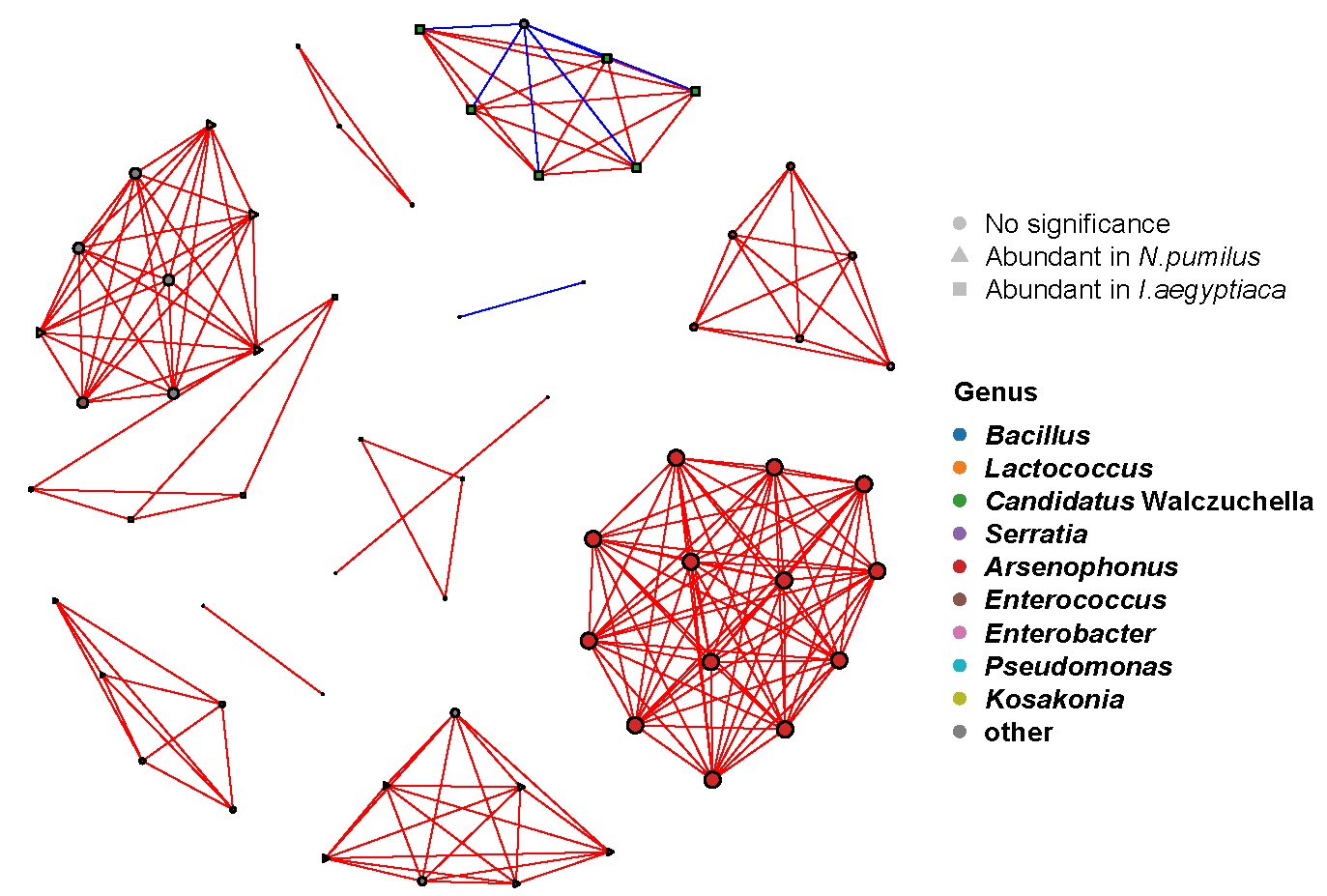


Fig. S7 The co-occurrence networks of the bacteria in the adult stage of *Icerya aegyptiaca*.


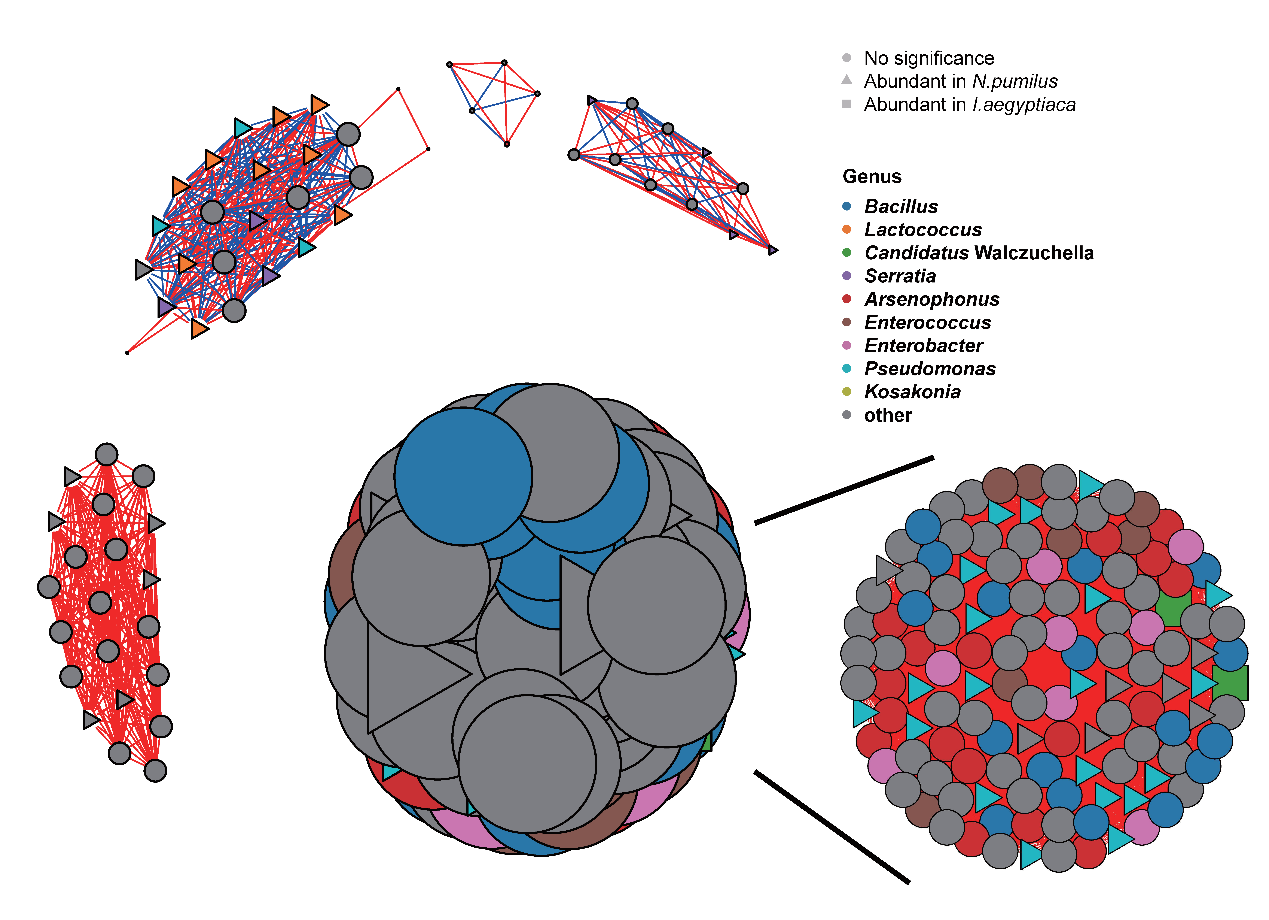


Fig. S8 The co-occurrence networks of the bacteria in the 1^st^ instar larvae stage of *N. pumilus*. The network on the right is the enlarged sub-network.


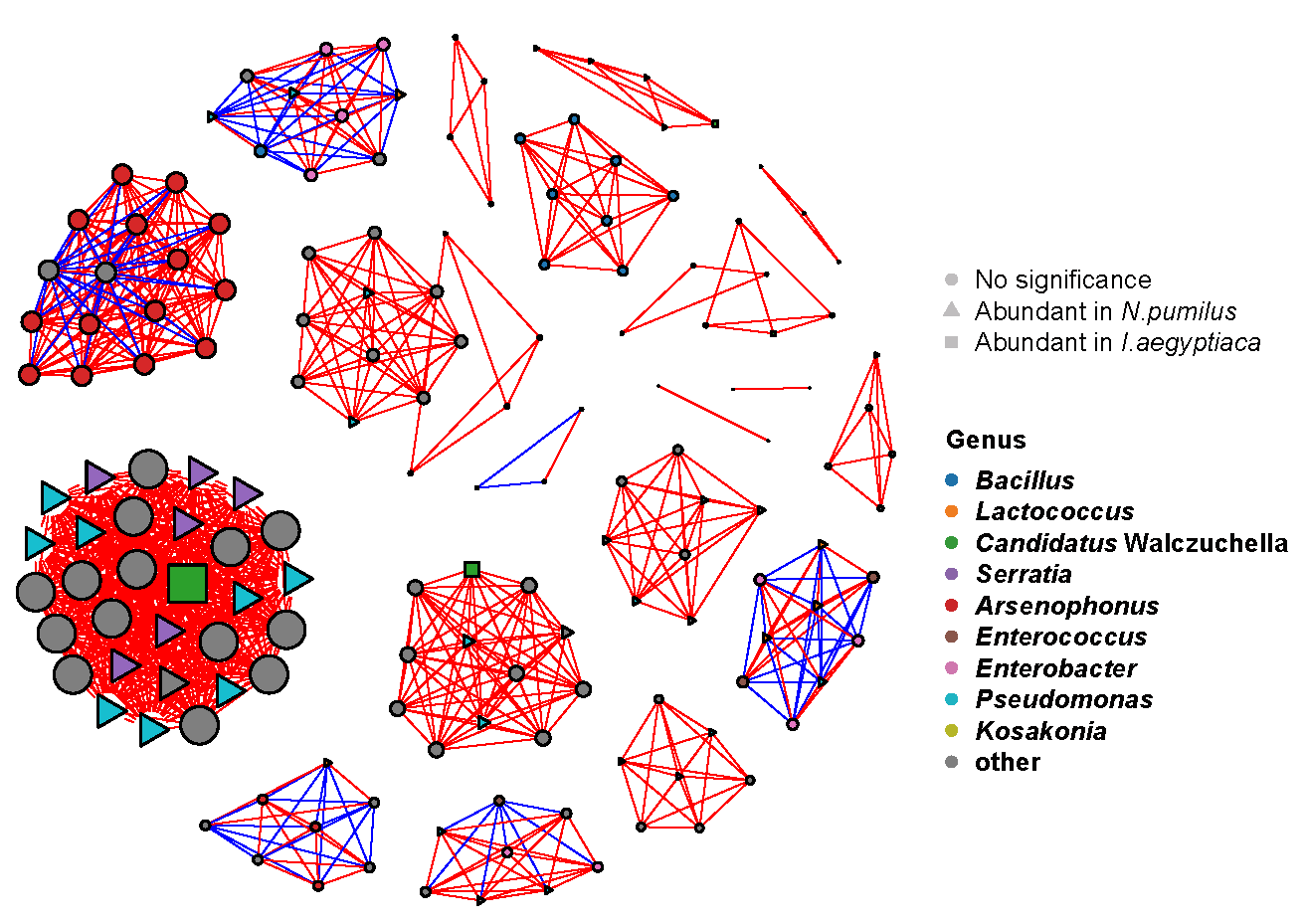


Fig. S9 The co-occurrence networks of the bacteria in the 3^rd^ instar larvae stage of *N. pumilus*.


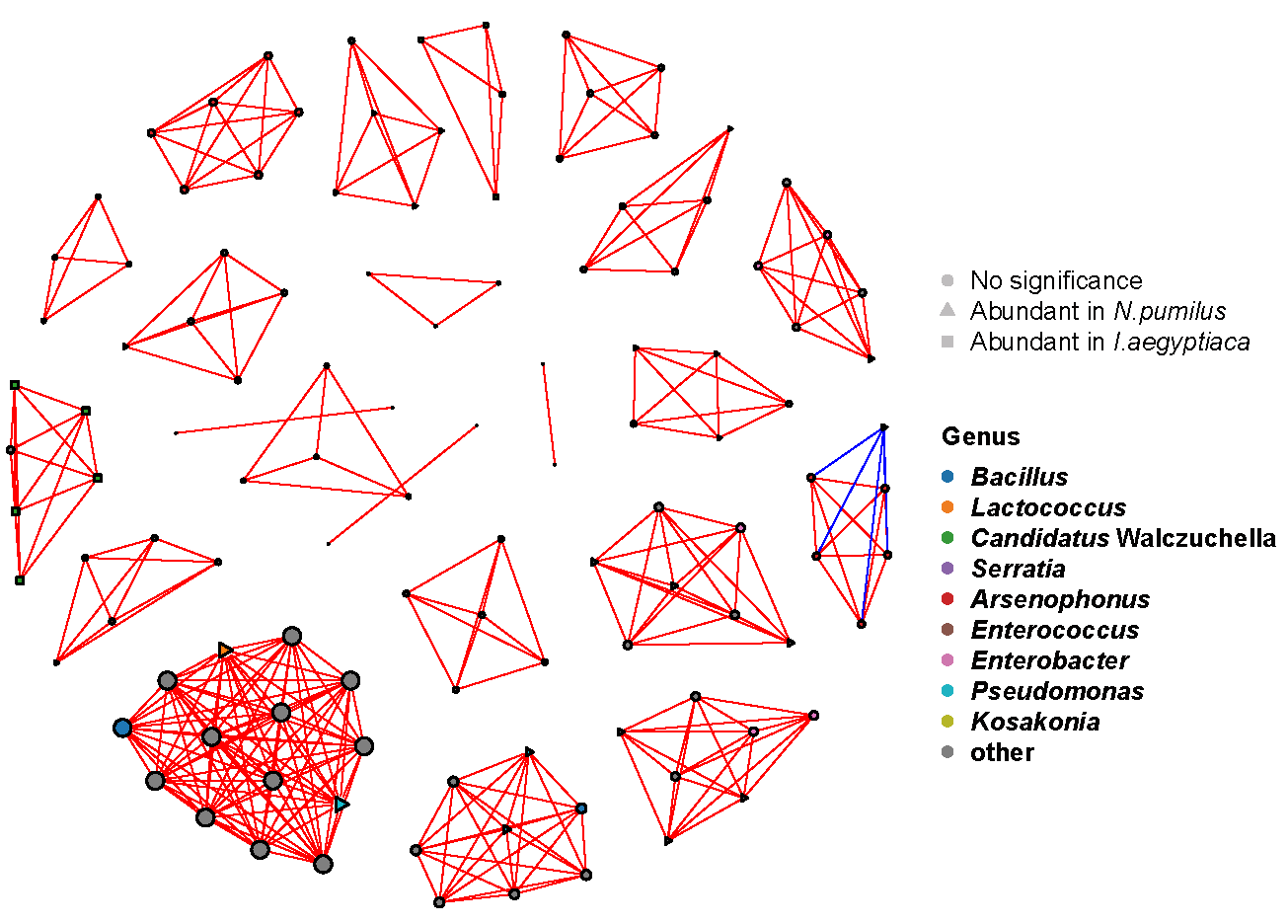


Fig. S10 The co-occurrence networks of the bacteria in the 4^th^ instar larvae stage of *N. pumilus*.


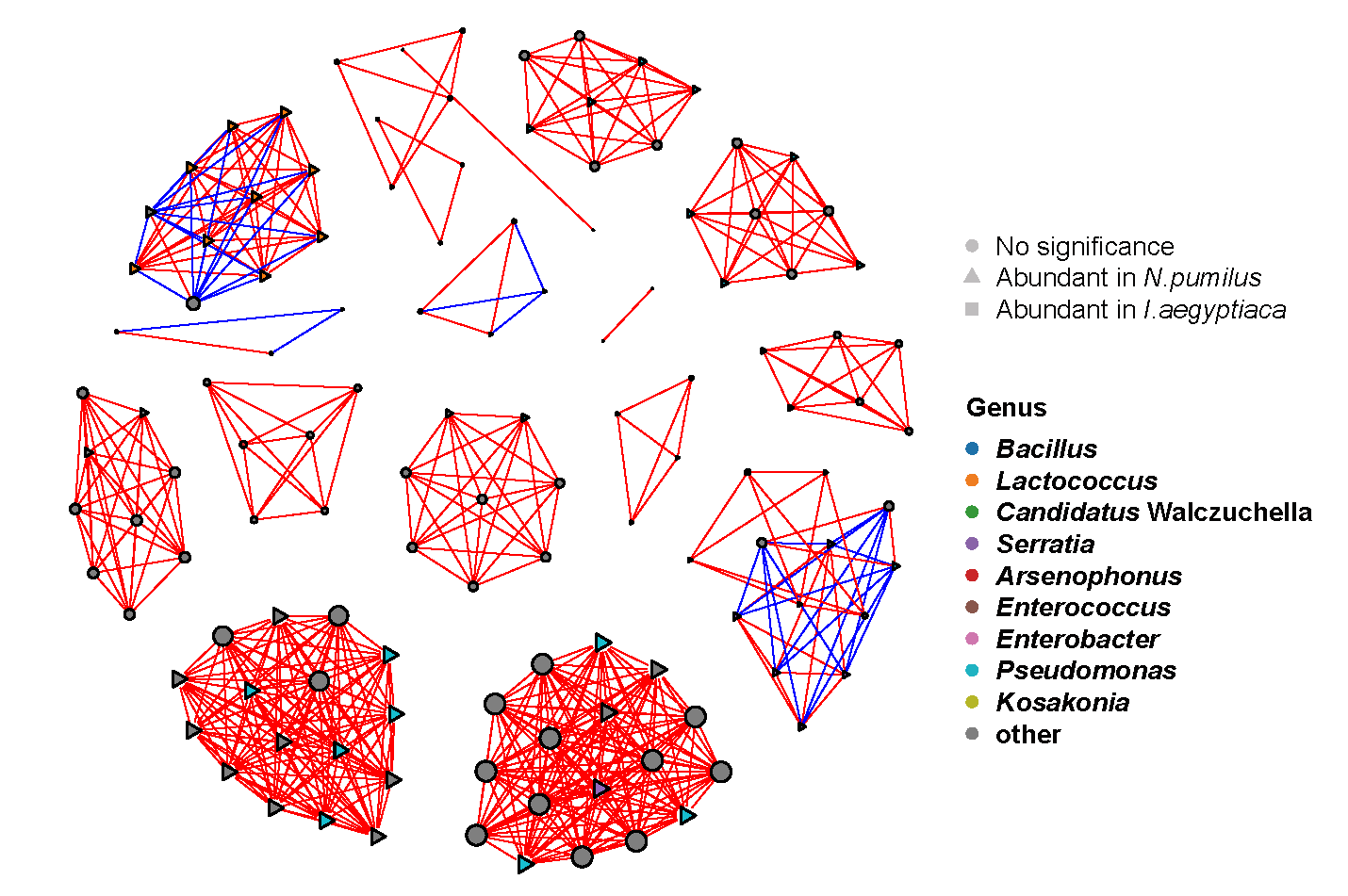


Fig. S11 The co-occurrence networks of the bacteria in the egg stage of *N. pumilus*.


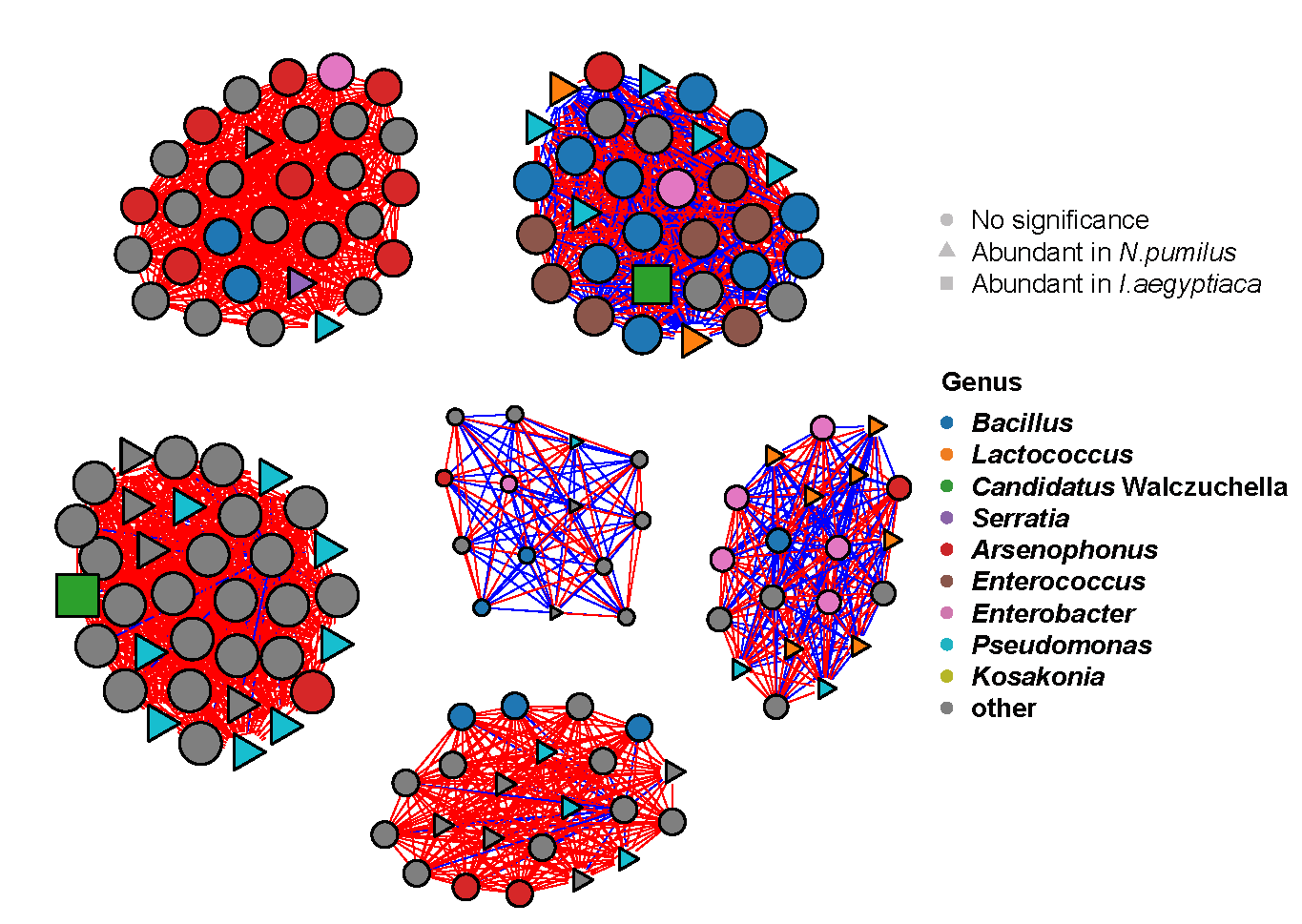


Fig. S12 The co-occurrence networks of the bacteria in the pupa stage of *N. pumilus*.


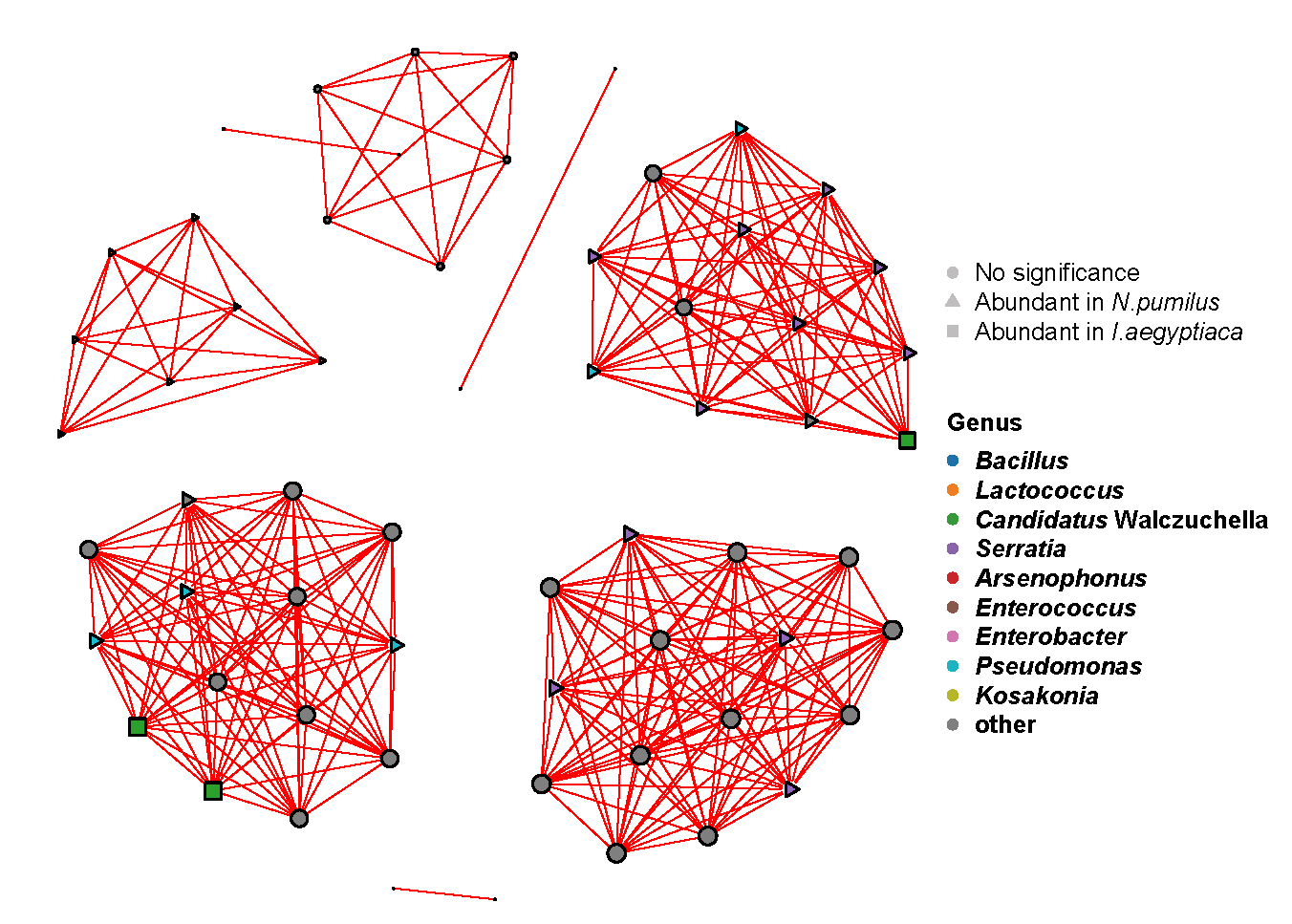


Fig. S13 The co-occurrence networks of the bacteria in the adult stage of *N. pumilus*.


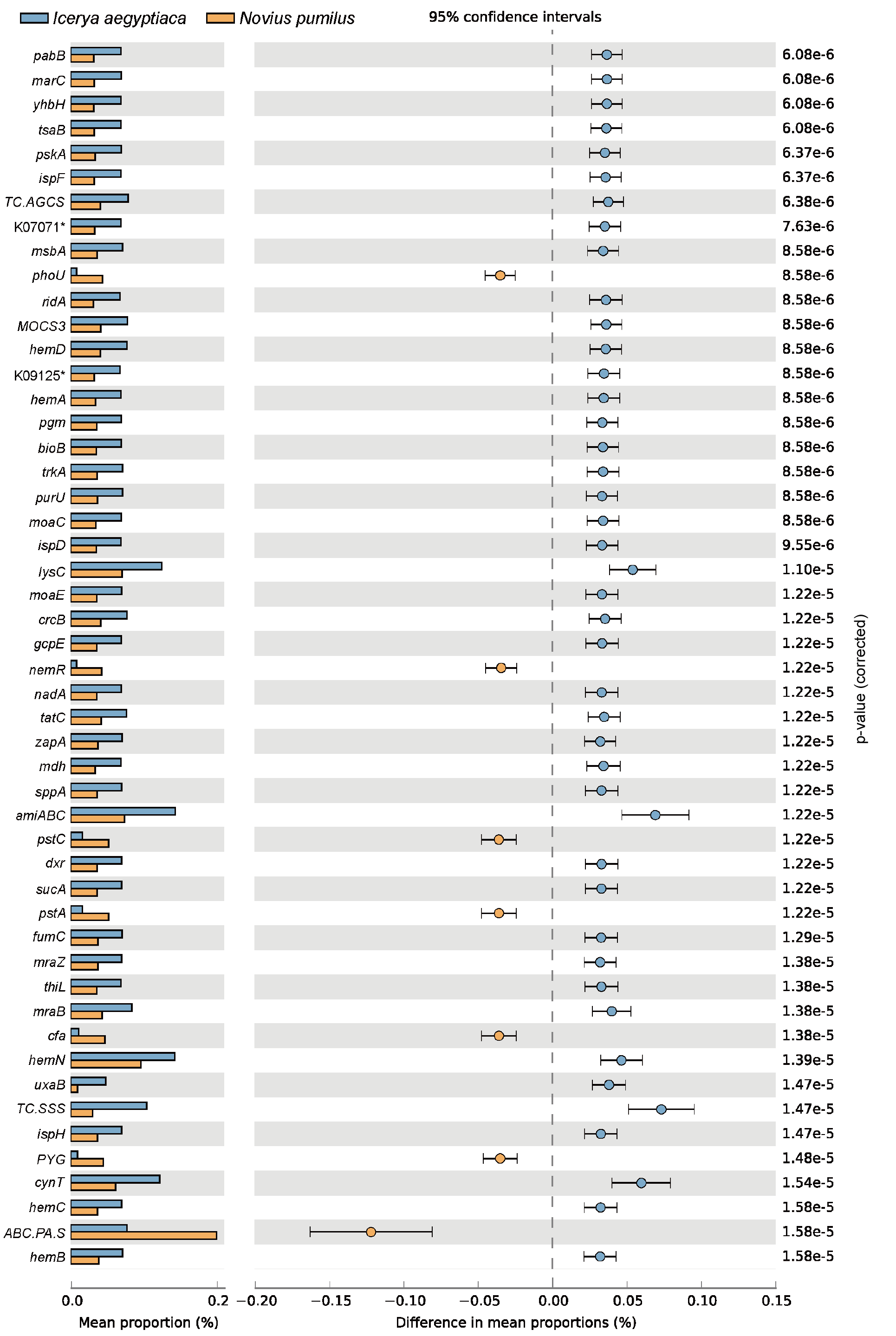


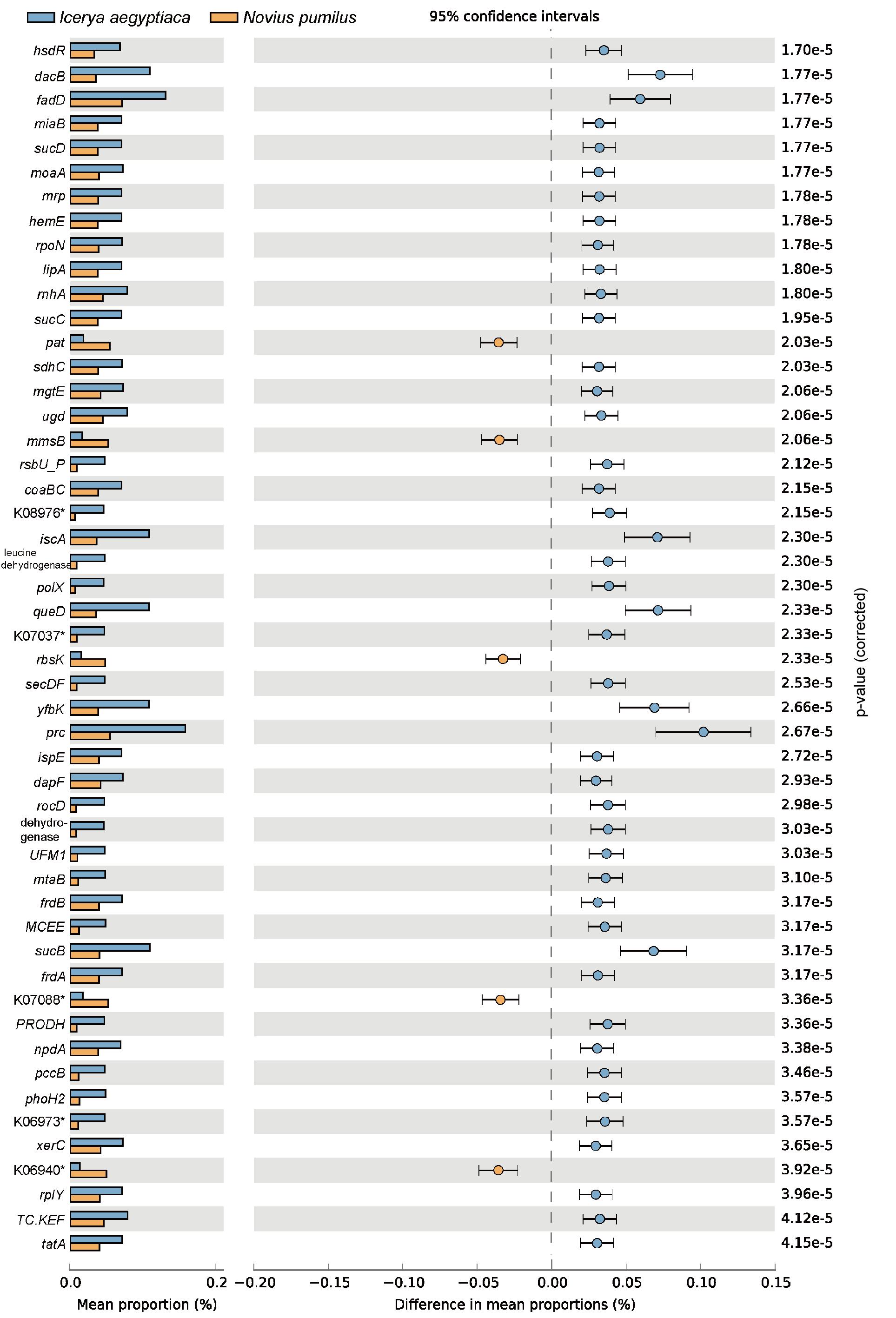


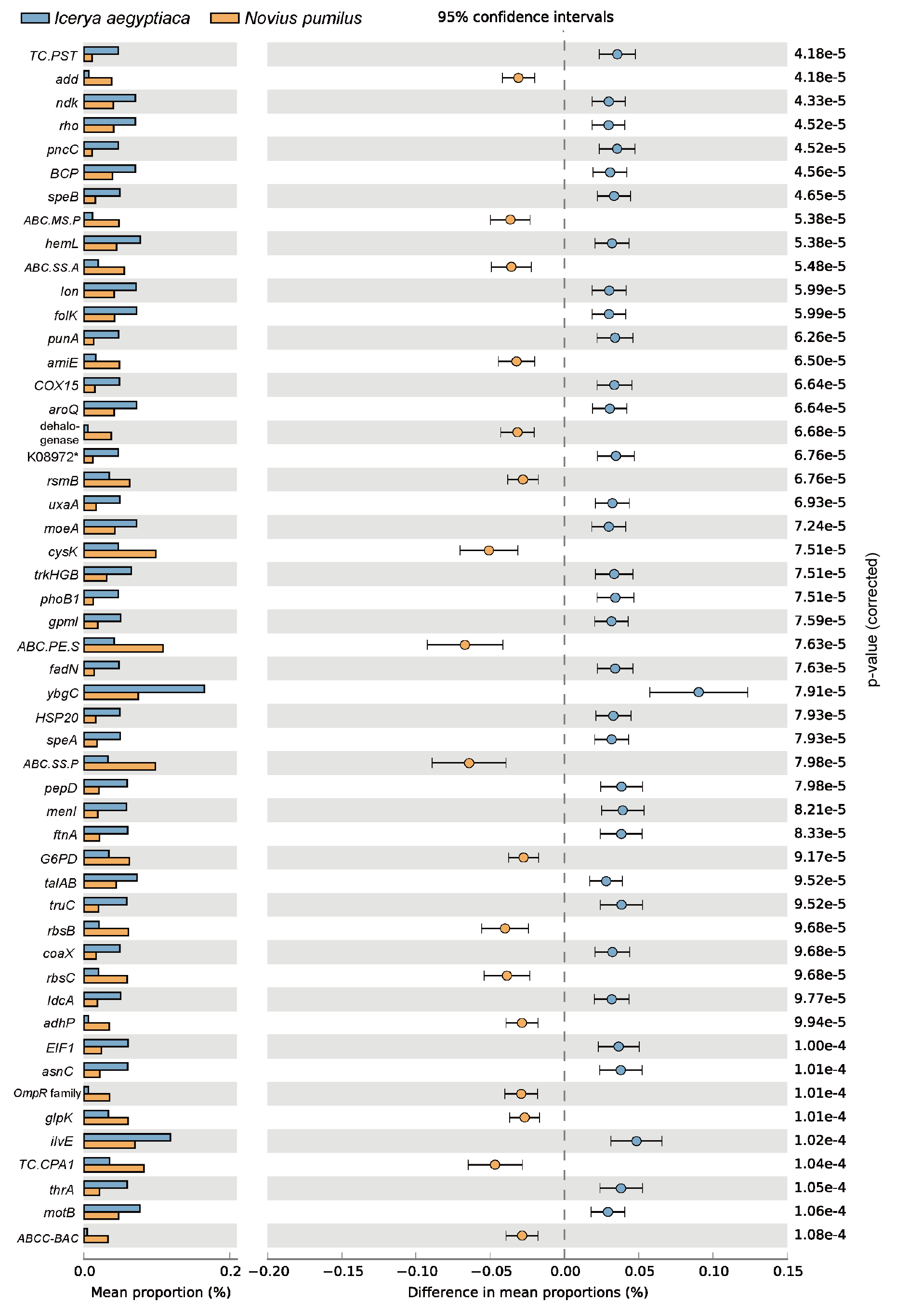


Fig. S14 The KEGG Orthologs significantly higher in *N. pumilus* or *I. aegyptiaca*.


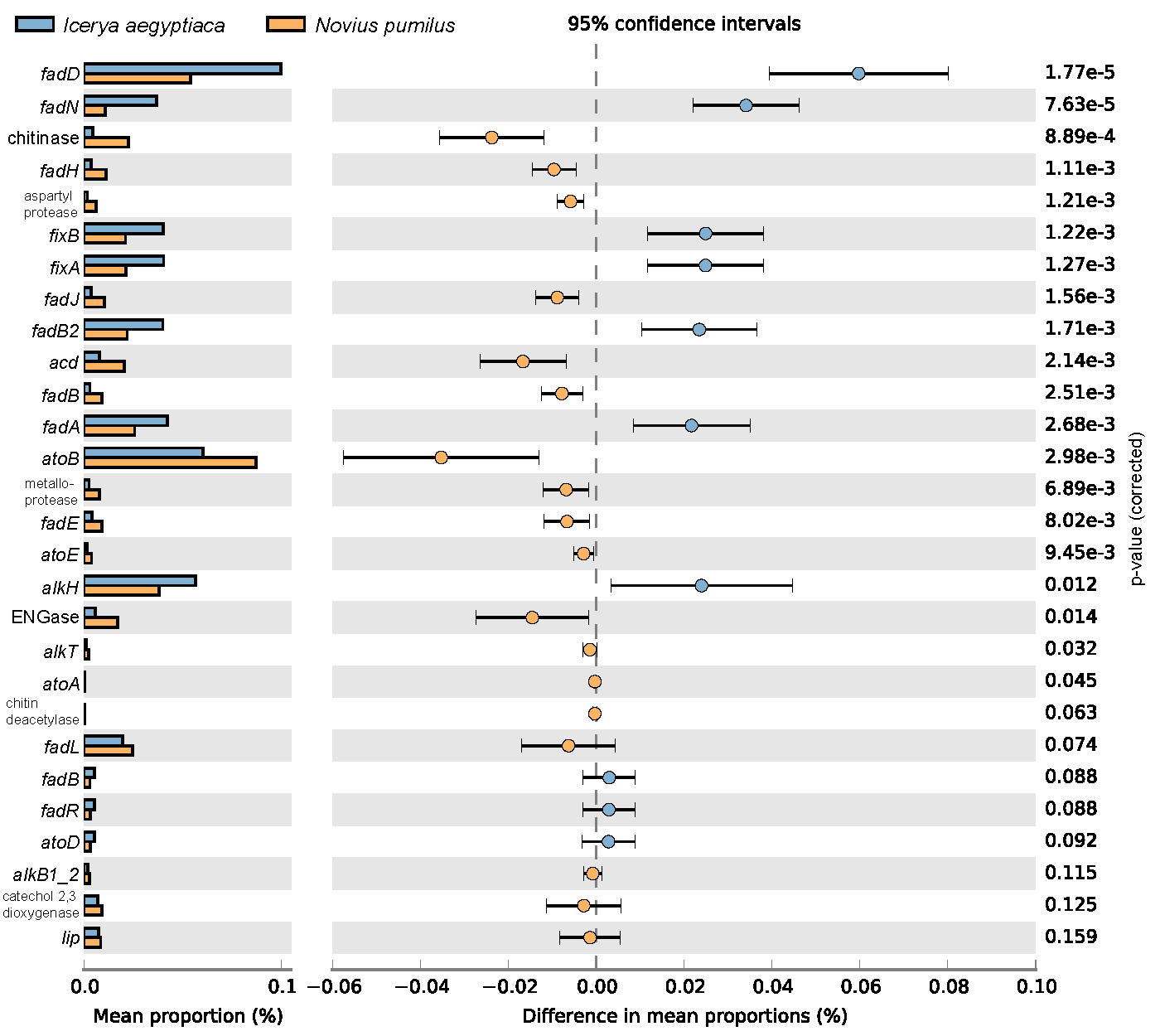


Fig. S15 The KEGG Orthologs of enzymes related to hydrocarbon, fatty acids and chitin degradation significantly higher in *N. pumilus* or *I. aegyptiaca*.
